# Supplementary material for: Structural Repetition Detector for multi-scale quantitative mapping of molecular complexes through microscopy
Source: Nat Commun. 2025 Jul 1;16:5767. doi: 10.1038/s41467-025-60709-1 (PMC12219329; doi:10.1038/s41467-025-60709-1)
Supplement: Supplementary file 1 — Supplementary Information [file 41467_2025_60709_MOESM1_ESM.pdf]

SUPPLEMENTARY INFORMATION

Supplementary Note 1: Theoretical foundation and core functionality of SReD

The Structural Repetition Detector (SReD) is an unsupervised computational framework designed to identify repetitive biological structures in microscopy images by exploiting local texture repetition. SReD operates by comparing local image regions (blocks) to detect recurring patterns without prior knowledge or constraints on the imaging modality. The algorithm's workflow includes the following preprocessing steps:

1. **Application of the Generalized Anscombe Transform (GAT):** This step stabilises noise variance, addressing the complex noise characteristics typical in microscopy images. The GAT employs a nonlinear remapping of pixel values to produce an output image with near-Gaussian noise and stabilised variance, preserving local contrast and overall image statistics (1). The pixel values of the transformed image are given by:

$$\tau_{GA}(Z_i) = \frac{2}{g_0}\sqrt{g_0 Z_i + \frac{3}{8}g_0^2 + e_{DC}} \qquad (S1)$$

   where g0 is the gain of the electronic system, eDC = σ2 − g0m, and m and σ2 are the mean and variance of the noise. The goal is to find parameter values such that the transformed image has variance as close as possible to 1. The initial parameter values can be user-provided or calculated automatically.

2. **Generation of a Relevance Mask:** This mask excludes regions lacking substantive structural information, based on local texture prominence quantified by variance or standard deviation. Due to the ubiquitous presence of noise, determining a threshold at which image texture is minimal becomes challenging. Instead of choosing an arbitrary value close to 0, we estimate the average noise variance of the input image using a robust estimator (2). This is obtained by sampling the variance of the input image using non-overlapping blocks of the same size as those used for the repetition analysis. The average noise variance is calculated by averaging the variance of the blocks below percentile 0.03. This variance threshold was identified as an optimal compromise: it minimizes the inclusion of structural information in the noise variance calculation while maximizing the number of blocks used, thereby improving the accuracy of the estimation The relevance threshold is defined by multiplying the estimated average noise variance (2) by an adjustable constant, with the default set at 0. The relevance threshold is established by multiplying the estimated average noise variance by an adjustable constant, with the default set at 0. This produces a binary mask outlining areas with sufficient structural content (Supplementary Fig. 1).

SReD's primary functionality revolves around two main analysis modes:

1. **Block Repetition Analysis:** The input image is probed for repetitions of a single reference block, which can be either simulated or extracted from the data. The output is a repetition map reflecting the likelihood of the reference pattern occurring at each location. The similarity score is computed using a correlation metric, which can be sensitive or insensitive to rotation.

2. **Global Repetition Analysis:** Every block in the input image is used as a reference, generating multiple repetition maps. These maps are integrated using an exponentially weighted average based on block similarity, producing a final map that reflects the relative frequency of each structural pattern across the entire image.

SReD provides different correlation metrics to perform repetition analysis. Most of these metrics are direct implementations of pre-established formulas (e.g., Pearson's correlation coefficient, Structural Similarity Index (SSIM), Normalised Root Mean Squared Error). Additionally, we introduced a rotation-invariant metric that mostly captures differences in local contrast – the "absolute difference of standard deviations" (ADSD). The ADSD is given by:

$$ADSD(X_i, Y_j) = |s_X - s_Y| \qquad (S2)$$

where X_i and Y_j are image blocks centered at pixels i and j, and s_X and s_Y are the standard deviations of image blocks X_i and Y_j.

The algorithm's output is a Structural Repetition Score (SRS) for each pixel, ranging from 0 to 1, which quantifies the degree of local structural repetition. This score can be interpreted as the likelihood of a specific pattern occurring at that location.

SReD also offers multiscale analysis capabilities by adjusting the ratio between the input image and block sizes, enabling the detection of structural patterns at various scales. This approach is particularly valuable for exploratory analysis of complex biological systems where the underlying structural patterns may not be fully known *a priori*.

Supplementary Note 2: Validation and application examples of SReD across diverse biological contexts

Detection of microtubules using simulated reference blocks. We evaluated SReD's ability to detect structures using simulated reference blocks. A series of blocks comprising lines and line crossings at different orientations were generated and used to detect microtubule structures in a STORM image reconstruction of a HeLa cell with labelled microtubules (3) (Supplementary Fig. 2a). Using the Pearson's correlation coefficient metric for orientation sensitivity, SReD produced repetition maps highlighting regions matching the simulated blocks (Supplementary Fig. 2b). The specificity was assessed by:

1. Detecting repetitions of a vertical line block in images with vertical lines at varying distances.

2. Detecting repetitions of an orthogonal line crossing block in images with crossings at different angles

In both cases, the average SRS decreased markedly when the specific structures were absent, demonstrating high specificity
(Supplementary Fig. 2c,d).

Detection of nuclear envelopes using empirical reference blocks. To demonstrate structure detection using empirical reference blocks, we extracted blocks containing nuclear envelope regions at various orientations from a DAPI-stained image of HeLa cell nuclei (4) (Supplementary Fig. 3a). Applying SReD with the Pearson correlation metric generated repetition maps highlighting regions matching the reference blocks, effectively mapping the nuclear envelopes and their local orientations (Supplementary Fig. 3b,c). This approach also enables the characterisation of morphological variations. We calculated the relative area percentage of each repetition map in the combined output to quantitatively describe nuclear shape (Supplementary Fig. 3d). These measurements distinguished different morphological states potentially related to cell division or stress.

Detection of HIV virus-like particles using Global Repetition. SReD's global repetition mode enables unbiased structure detection by quantifying the relative repetition of all structures in an image. We analysed an image of a Jurkat cell expressing na HIV Gag-EGFP construct that induces virus-like particle (VLP) assembly (Supplementary Fig. 4a,b). The global repetition map, calculated using the absolute difference of standard deviations, showed:

- The most repetitive structure was the cell background signal (diffusing Gag protein).

- Lower SRS structures corresponded to active VLP assembly sites.

- Some structures not discernible in the input image were detected, likely early VLP assembly stages.

We identified VLP locations by calculating local maxima. The repetition map provided more detections than the input image when using the same prominence threshold (Supplementary Fig. 4c).

Multiscale detection of Nuclear Pore Complex structures. SReD's multiscale analysis capability was demonstrated by examining nuclear pore complexes (NPCs) in STORM reconstructions of labelled gp210 proteins. Global repetition maps were calculated at different scales by modifying block-to-image size ratios (Supplementary Fig. 5a,b):

1. Original image (400x400 pixels), 5x5 pixel blocks: detected single nucleoporins.

2. Original image, 15x15 pixel blocks: detected nucleoporin clusters.

3. Downscaled image (200x200 pixels), 25x25 pixel blocks: detected entire NPC units.

This demonstrates SReD's ability to detect structures at multiple scales without requiring structural priors.

Supplementary Note 3: Detection of HIV particles in transmission electron microscopy data

SReD's versatile structure detection applies across diverse microscopy modalities. We demonstrate this feature using SReD data to identify HIV viral particles in transmission electron microscopy (TEM) data (Supplementary Fig. 6; Supplementary Movie 1). The data included manually annotated particle counts, positions, and maturation states, as well as results from a deep learning-based detection approach (5).

Pre-processing data. The raw TEM image reconstruction was pre-processed to optimize computational efficiency and improve feature detection (Supplementary Fig. 6a). First, the image was blurred using a Gaussian kernel (sigma = 2 px) and downscaled to 25% of its original size to improve the computational efficiency of further analyses. The downscaled image was subsequently inverted to enhance structural contrast (this step is optional). Then, the image was processed according to the specific structures being analysed. To analyse particles containing mature capsids, a Median filter with a radius of 5 pixels was applied. For particles of all types, an additional blurring step was performed using a Gaussian kernel (sigma = 10 px).

Block repetition analysis. A structural reference block was extracted from each processed image and SReD was then used to generate block repetition maps with the Pearson correlation coefficient metric (Supplementary Fig. 6b,c). To emphasize features of interest, the repetition maps were nonlinearly remapped using a power transformation with exponent 5. The final image reconstruction featuring the input data and the SReD repetition maps was generated by overlaying the different channels using the ImageJ "Merge Channels" function (Supplementary Fig. 6d).

Particle localization and comparison with existing methods. Structure localisation in each repetition map was achieved by applying Otsu's thresholding method (6) to the repetition maps, enabling the identification of particle positions and counts using the ImageJ/Fiji "Analyze particles" function with default settings. Whereas the original study (5) identified 37 particles (34 mature and 3 immature), our approach detected 58 particles, of which 34 were identified as mature. The difference in total particle count is attributable to methodological differences - while the original study focused exclusively on mature and immature viral particles, our approach identified all repetitive structures, including non-specific artifacts and damaged particles. Despite detecting additional structures, our results closely matched the mature particle counts reported in the original study (5). To validate the specificity of SReD, we compared the spatial positions of our detections with those reported in the original annotations. Approximately 90% of the particles identified by SReD overlapped with the annotated positions.

These results demonstrate the robustness and specificity of SReD in detecting viral structures. Moreover, they highlight its generalizability to microscopy modalities beyond fluorescence microscopy, such as EM.

Supplementary Note 4: Detection and characterization of HIV assembly platforms using STORM data

The spatial organization of viral components is crucial for understanding viral assembly mechanisms. Given that viruses' sizes are often below the diffraction limit of light, super-resolution microscopy provides a warranted approach for analysing viral structures with high precision. We analysed STORM data featuring Jurkat cells expressing an HIV Gag(i)GFP construct, treated with either DMSO (vehicle control) or CK666, an Arp2/3 complex inhibitor that induces actin debranching (7).

Generation of image reconstructions. The original list of localisations (7) was processed using ThunderSTORM (8). Initial filtering excluded localisations with a standard deviation ("sigma") below 25 nm or above 250 nm. A density filter with a distance radius of 50 nm was then applied, followed by removal of duplicate localisations, and merging of repeating molecules within 20 nm. Drift correction was performed using ThunderSTORM's "fiducial markers" module. Processed localization datasets were subsequently used to generate image reconstructions with the "Normalized Gaussian" visualization method, applying a magnification factor of 15, yielding a final pixel size of 10.7 nm (Supplementary Fig. 7a, top left halves). Background signal was removed by masking, using a Gaussian kernel with (sigma=100), followed by Otsu thresholding (6).

Detection and quantitative analysis of viral assembly platforms. The study that provided the datasets used here reported an average Gag cluster size of approximately 100 nm, in the same range than HIV infected T-cells that did not experience any treatment (7). Based on this observation, SReD was used to detect repetitions of a reference block containing a 100 nm particle extracted from the control image (Supplementary Fig. 7a). Local maxima in the repetition maps were identified using ImageJ's "Find Maxima" function with a prominence threshold of 0.1. For comparison, local maxima were also extracted from the input image reconstructions after normalizing to the same range as the repetition maps (i.e., [0,1]). In agreement with Dibsy *et al.* (7), a higher number of particles was detected in the CK666-treated sample compared to the DMSO control (Supplementary Fig. 7b; Input: 193 (DMSO) vs. 403 (CK666); SReD: 2273 (DMSO) vs. 3084 (CK666)). Notably, a substantially higher number of particles were detected in the SReD repetition maps compared to the input images (Supplementary Fig. 7b). These detections followed the same trend observed in those from the input images, with CK666-treated samples exhibiting more structures than the DMSO control. These results demonstrate that SReD repetition maps provide an improved platform for structure detection. Furthermore, block repetition analysis provided insights into the stability of different viral assembly platform morphological states, as evidenced by the significantly different SRS distributions of the DMSO and CK666 platforms (Supplementary Fig. 7c,d; $p=1.9\times10^{-14}$; Mann-Whitney U-test).

These findings highlight the impact of actin network dynamics on HIV assembly and demonstrate that SReD-based analysis offers a powerful approach for detecting and characterising viral structures with enhanced sensitivity.

Supplementary Note 5: Automated analysis pipeline for spectrin ring detection in neuronal axons

Automatic estimation of axon orientations. A robust analysis of ring periodicity using autocorrelation functions requires the axon segments to be oriented with their long axis parallel to the horizontal axis. We designed a streamlined approach that uses SReD's block repetition to estimate the orientation of the axons along their span (Supplementary Fig. 8a). This information can be used for downstream applications, such as estimating the distribution of angles in each sample (Supplementary Fig. 8b,c) or automatic region extraction and rotation. Our method is fully implemented in ImageJ macros and works as follows:

1. **Calculation of the axons' "skeletons". "**Skeletonize" function produces a binary mask of the axons' skeletons by shrinking their to a 1-pixel wide line along their centers of mass. To improve the results of this function, the images are preprocessed by applying a Gaussian blur (sigma=15 px) to retrieve only the high-order structures and discard the single-molecule low-order information. Then, thresholding is performed using Otsu's method (6) to remove unwanted objects. The "Skeletonize" function is applied to the thresholded images. A Gaussian blur (sigma=2 px) is applied to the skeletons to avoid diagonal discontinuities derived from the 1 pixel-wide lines that form the skeletons. Finally, a range normalisation step is performed to bring all the skeletons to the same intensity interval.

2. **Generation of synthetic blocks comprising lines at different orientations.** This is done by designing a block containing a vertical 1 pixel-wide line in a 90x90 black canvas. Then, copies of this block are created and rotated in 10º increments, until all possible orientations are recapitulated at that angular resolution. A Gaussian blur (sigma=2 px) is applied to the blocks to match the appearance of the blurred axon skeletons. The synthetic blocks are then cropped into 45x45 pixel blocks to avoid border artefacts and normalised to their intensity range.

3. **Calculation of block repetition maps using SReD.** SReD's block repetition is used to generate repetition maps using the synthetic blocks and axon skeletons generated previously. Each round of block repetition produces a repetition map where regions containing skeletons at the specific orientations display higher SRS. The repetitions maps are normalised to their intensity range, multiplied by the Gaussian blurred masks to remove unwanted detections, and normalised once more.

4. **Generation of the angle maps.** Each coordinate of the 1 pixel-wide skeletons is labelled with the angle corresponding to the highest SRS in the repetition maps.

Reference block optimisation for ring pattern detection. The detection of specific structures requires the use of a reference. In SReD, the reference is provided as an image block containing a representation of the structure of interest. To minimise the bias of downstream analyses towards the reference block, we devised a method to optimise the reference block based on the data characteristics (Supplementary Fig.

9a). The method is implemented as a combination of ImageJ macros and Python code. It works as follows:

1. **Conceptualisation of the reference block.** The reference block used for spectrin ring detection should recapitulate a periodic ring pattern while using the minimum amount of information to minimise bias towards that pattern's characteristics. We concluded that the simplest pattern would comprise a black canvas with 3 vertical lines resembling a side-view of a 2D projection of rings. While using 2 lines instead of 3 seems more parsimonious, doing so would centre the pattern at the inter-ring spacing and not the ring itself. The 3-ring pattern can be defined by two parameters: (i) inter-ring spacing and (ii) ring height. The first parameter is the main target of our study, while the latter is less important and is mostly a function of axon girth.

2. **Optimisation of the ring pattern's parameters.** To minimise the bias of our study towards the reference pattern, a parameter sweep was performed to optimise the pattern's characteristics according to the input data. To do this, we generated a collection of 248 blocks incorporating various combinations of inter-ring spacing and ring height. Representative segments of distal axons (6 for each group) were extracted from the data. From these axon segments, a total of 30 test regions were extracted and rotated to align with the horizontal axis. This rotational adjustment guaranteed consistency when applying the same set of reference blocks across the datasets, eliminating any potential variations stemming from block rotation and interpolation. SReD was used to calculate block repetition maps for every reference block and the autocorrelation functions of the repetition maps was calculated. The relative amplitude of the autocorrelations' first harmonic was used to assess how effectively each block captured the underlying periodic pattern. We systematically identified the set of block parameter values that maximised the first harmonic's relative amplitude (Supplementary Fig. 9b-d). This optimised set of parameter values serves as a reliable representation of the periodic pattern within the dataset. The optimisation was performed separately for each dataset analysed in this study.

Detection of ring patterns in large fields-of-view. Using the optimised ring pattern and the angle distributions previously generated, SReD can be used to map ring patterns at all orientations across large fields-of-view.

1. **Preparing the input data for analysis.** An input image comprising a large-field-of-view of axons featuring labelled spectrin rings is copied and rotated for each angle defined previously (0º to 180º in 10º steps). To avoid cropping at the borders, the image is zero-padded before this step. A range normalisation is also applied.

2. **Detecting spectrin rings at all orientations.** Block repetition maps are calculated for each rotated input. Doing this ensures that each axonal region in the input is probed at least once while oriented parallel to the horizontal axis. Then, the repetition maps are rotated back to their original orientation.

3. **Generation of angular weight maps.** From the repetition maps calculated previously, we are interested in retrieving only the information that is relevant for a specific angle. For example, in the repetition map calculated from the input image rotated by -10º, we want to keep the segments whose orientation relative to the horizontal axis was estimated to be 10º. To do this, we use a weight multiplication approach. An angular weight map is generated for each orientation by transforming the angle repetition maps so that they accommodate the full extent of the axons in the input data instead of just their center line. The transformation consists of a Gaussian blur (sigma=10 px) and a power function (exponent of 8), followed by a range normalisation step.

4. **Application of the angular weight maps and generation of the final output.** The angular weight maps are multiplied by the ring repetition maps that were calculated using inputs rotated to their corresponding angle. For example, the ring repetition map calculated from the input image rotated 10º is multiplied by the angular weight map comprising regions that are oriented at 10º from the horizontal axis. This step retains only the ring pattern information that is relevant at each orientation. After a range normalisation step, the final image reconstruction is generated by averaging the weighted repetition maps (Fig. 2b).

Automatic extraction and quantitative analysis of ring patterns in axon segments. Previous studies focusing on characterising the periodicity of spectrin rings in axons were limited by the need to manually select regions for analysis. This method is labour-intensive, and the results can vary between experts. Semi-automatic methods also exist, where algorithms such as NeuronJ (12) produce tracings of the neurons and provide a framework for quantitative analysis. However, these algorithms do not consider the underlying low-order structures, resulting in axon segments that may or may not contain periodic ring patterns, which impacts their quantitative descriptions. Our approach leverages SReD's multiscale capabilities to ensure that only regions containing patterns are analysed:

1. **Detection of high-order ring patterns.** Ensuring that the axon segments analysed contain ring patterns is essential to avoid corrupting the results with unwanted structural patterns. This entails mapping regions of the input data where high-order patterns exist. This is often achieved by convolving data with Gaussian kernels. However, this method inherently discards the low-order information, and its results are mostly a function of local signal intensity. We approach this problem by leveraging SReD's multiscale analysis capabilities. Using the optimised ring reference block, a new reference block comprising an extension of the original is generated with 9 rings instead of 3 (Supplementary Fig. 10a). SReD is used to calculate block repetition maps, in which regions where the high-order patterns are more likely to exist will have higher SRS (Supplementary Fig. 10b). A quantitative comparison between our approach and the more common approach using convolutions with Gaussian kernels demonstrates that SReD produce a more robust analysis by retaining the low-order information of ring units while providing mapping the presence of high-order patterns (Supplementary Fig. 10c,d). The high-order repetition map is

thresholded using the Otsu method (6) to generate a binary mask where regions containing high-order patterns are segmented. These regions can be used for downstream analysis using the optimised low-order reference block, ensuring that the data analysed contains the structures of interest.

2. **Removing axon crossings and bifurcations.** This step is essential to the study of axon ring periodicity because (i) regions containing axon crossings may contain multiple periodic patterns at different phases and orientations, and (ii) the arrangement of spectrin in regions where axons bifurcate is not well-characterised and is known to not display the periodic patterns observed in more distal regions (13). These regions are filtered out from the analysis using a custom ImageJ macro that detects coordinates where a pixel of the skeleton contacts with more than 2 pixels. This creates discontinuities in the skeletons exactly where the crossings/bifurcations are detected. Then, the extremities of the isolated segments are iteratively pruned, until each extremity is reduced by a number of pixels equal to the radius of the regions chosen for downstream analysis (75 pixels). This eliminates unwanted regions.

3. **Extraction of regions containing periodic patterns from the input data.** The segmented axon skeletons generated in the previous step are used as a basis to extract structurally relevant regions (Supplementary Fig. 11a). The characteristics of the segments already provide a basis to study the stability of spectrin rings, enabling comparisons between control axons and axons treated with swinholide A. We demonstrate this by calculating their length distribution. No significant differences were found between the length distributions, indicating that swinholide A treatment did not impact the high-order arrangement of spectrin rings (Supplementary Fig. 11b). To automatically extract regions for the analysis of low-order patterns, the axon segments are divided into non-overlapping 150x150 pixel blocks centred at their skeletons. The coordinates of each block are used to crop the corresponding regions from the large field-of-view ring repetition maps generated previously. Then, each block is rotated to become parallel to the horizontal axis using the angle estimations calculated previously. This process yields a library of regions containing ring patterns and their ring repetition maps (Supplementary Fig. 11c).

4. **Quantitative analysis of local ring patterns.** The rings patterns detected and extracted using SReD can be used for downstream analysis. This enables the characterisation of the patterns' characteristics. We calculated the autocorrelation functions of each region and, from the first harmonics' position, determined that the average inter-ring spacing in our datasets was approximately 180 nm. We found no significant differences between the control and the swinholide A-treated samples (Supplementary Fig. 11d). The first harmonics' amplitude can be interpreted as the strength or prominence of the periodic pattern. Here, we found a 12% reduction in prominence in the samples treated with swinholide A (Supplementary Fig. 11e). This reduction was smaller compared to previous studies (1). However, when we analysed the fraction of regions containing periodic patterns, we found a 39% reduction in the samples treated with swinholide A that was not reported previously. These results

demonstrate that our approach enabled discerning the effects of swinholide A in pattern frequency from pattern prominence.

5. **Evaluation of SReD's performance in noisy data.** The image reconstructions used in the previous sections were generated from localisation data obtained using Single-Molecule Localisation Microscopy (SMLM). Due to the synthetic nature of these reconstructions, the images produced are devoid of noise originated from the electronic acquisition systems. However, the generalisability of our method to other microscopy modalities requires sensitivity to structures in noisy data. We evaluate SReD's performance in noisy data using a test image of an axon segment, to which Gaussian noise with incrementally higher standard deviation is added. SReD is then used to detect repetitions of the optimised ring block, and its performance is evaluated by comparing the block repetition maps with the "noise-free" control sample (Supplementary Fig. 12a). The repetition maps calculated showed that SReD was able to detect ring patterns in a wide range of signal-to-noise ratios (SNRs). Notably, it enabled detecting ring patterns in images with very low SNR, where structures are usually not discernible (Supplementary Fig. 12b). Despite the reduction in confidence, evidenced by the lower SRSs, the detections remained specific, as shown by their colocalisation with the reference structures (Supplementary Fig. 12b, bottom row). We quantified the performance of the algorithm by calculating correlation metrics between the noisy inputs and the control (Supplementary Fig. 12c). We used two metrics commonly used to assess image quality and fidelity (the Structural Similarity Index Measure (SSIM) and the Root Means Squared Error (RMSE)). This analysis showed that the repetition maps were a more robust representation of the control sample. We also analysed the results using autocorrelation functions. Here, the autocorrelation harmonics degraded quickly in the noisy input images while remaining well-defined in the repetition maps (Supplementary Fig. 12d). The characteristics of the autocorrelations' harmonics revealed that the expected inter-ring spacing of approximately 190 nm was discernible in all repetition maps, while in the input data it was only detected in the control sample. The prominence of the periodic patterns attributes high confidence to these conclusions (Supplementary Fig. 12e). Together, our results show that SReD's repetition maps were a superior platform for the quantitative analysis of periodic patterns and structure detection in noisy data when compared to the direct analysis of input data.

6. **Evaluation of SReD's specificity.** When detecting repetitions of a reference structure, it is important to evaluate their specificity, since a common caveat of this approach is the introduction of bias towards the reference. This could result in false-positive detections. We evaluated SReD's specificity by analysing an image of axon segment whose width was incrementally stretched, disrupting the periodic ring pattern's characteristics (Supplementary Fig. 13a). SReD was used to calculate repetition maps using the optimised reference block (Supplementary Fig. 13b). The average SRS across the repetition maps decreased abruptly upon stretching the input image, indicating a high specificity for the reference structure (Supplementary Fig. 13c). Importantly, this decrease in the average SRS was followed by a slight increase that then faded, suggesting the detection of a secondary pattern when the input image was

stretched to twice the original width. This observation was investigated by analysing the corresponding repetition maps. We determined that the secondary pattern corresponded to the detection of the second harmonic of the ring periodic pattern. This was explained by the alignment of the reference block's centre line with a single spectrin ring or the two outer lines with two spectrin rings (Supplementary Fig. 13d,e). The confidence of this detection was highest at a stretch factor of 2 because the patterns period is exactly twice the original at this level. Furthermore, autocorrelation functions showed that, in these conditions, the peak of the second harmonic colocalised with the input's intrinsic pattern (Supplementary Fig. 13f). Analysis of the first harmonic of the autocorrelations revealed that the period of the pattern in the input image increased with the stretch factor, whereas in the repetition maps it remained at approximately 180 nm but with incrementally lower prominence, indicating the high specificity of the algorithm. The second harmonic remained at twice the period of the first harmonic while the reference pattern was present and reflected the input's intrinsic period thereafter (Supplementary Fig. 13g,h). Together, these results suggest that SReD is highly specific for the reference structures, with false-positive detections still reflecting the structural arrangements of the input data and being discernible from true-positives by the magnitude of the SRS.

Supplementary Note 6: Evaluation of SReD's performance in images corrupted with non-specific structures

Generating images containing non-specific structures. Due to non-specific labelling and autofluorescence, microscopy data often contains unwanted structures, corrupting its integrity. This presents a challenge for structural analysis. Unlike camera noise, which can be effectively addressed through established denoising algorithms leveraging its predictable characteristics, non-specific structures add complexity by introducing additional structures into the data. This can obscure specific structures or alter their appearance, complicating accurate analysis. While an expert might visually distinguish specific from non-specific structures in certain cases, their presence still impacts the analysis. Given that SReD's global repetition mode is designed to detect all structures in an image, it was crucial to evaluate how non-specific structures affect the analysis of specific structures in a controlled sample. To achieve this, non-specific structures were introduced into images of a Jurkat cell producing virus-like particles (VLPs), along with simulated free particles exhibiting similar characteristics (Supplementary Fig. 14a). Perlin noise at varying frequencies was added to simulate non-specific structures with various levels of complexity using the Python (3.9.4) "Perlin-Noise" library (v1.13). Different noise frequencies were achieved by modifying the "scale" parameter, which corresponds to the inverse of the frequency. Higher frequencies generate structures with increased complexity.

Analysis of SReD's robustness against non-specific structures. Global repetition maps were calculated for each sample (Supplementary Fig. 14b). The coordinates of small round objects were identified by calculating 3D local maxima. The number of detections in the global repetition maps was higher than in the input images for all samples (Supplementary Fig. 14c). Specifically, the input images had detection counts of 251, 235, and 223 at noise frequencies 0, 0.02 and 0.04, while the global repetition maps showed detection counts of 505, 281, and 462 (Supplementary Fig. 14d). Furthermore, the percentage of ground-truth detections was substantially higher in the global repetition maps compared to the input images. In the input images, the ground-truth detection percentages were 32%, 30%, and 24%, whereas in the global repetition maps, these percentages increased to 96%, 63%, and 79%, respectively (Supplementary Fig. 14e). These results underscore the significant impact of non-specific structures on structural analysis and highlight the enhanced detection capabilities of SReD's global repetition mode. By substantially increasing the detection of ground-truth structures, even in the presence of complex non-specific noise, this method demonstrates its robustness and effectiveness in accurately identifying specific structures within corrupted microscopy data.

Supplementary Note 7: Assessment of the microtubule network's stability along time using SReD

Calculation of spatiotemporal global repetition maps. The versatility of SReD's global repetition mode is demonstrated by extending its 3D capabilities to enable spatiotemporal analysis. A 2D time-lapse was analysed by defining time as the third dimension, resulting in the analysis of texture repetitions within a given time interval. Several time intervals were defined, and subsets of the input data were created by only keeping images within those intervals. Then, the depth of SReD's block size was defined as having the same size as the number of images in each subset. Since SReD's sampling scheme dictates that blocks are not allowed to analyse regions beyond the data's dimensions, this results in a single repetition map for each subset, which highlights the degree of repetition of local textures across the specified time intervals (Supplementary Fig. 15a). Here, the global SRSs can be interpreted as the spatiotemporal stability of structures at each coordinate, with higher SRSs representing more stable structures and vice versa.

Analysis of microtubule dynamics. Assessment of microtubule dynamics was conducted using spatiotemporal global repetition maps. These maps were generated by comparing the state of structures at each time interval with their initial state, employing NRMSE metrics (Supplementary Fig. 15a). Higher NRMSE values indicated less stability of structures over time, and vice versa. NRMSE maps derived from SReD's repetition maps were compared to maps derived from (i) final images of each time interval and (ii) temporal projections. Visual analysis revealed that NRMSE maps from input images highlighted displacements between successive time points. Some regions with high error values in these maps corresponded to local noise patterns because the block size required to capture structures was unable to capture the average noise distribution. NRMSE maps from temporal projections, due to their additive nature, were substantially corrupted by local noise differences. Consequently, these maps poorly distinguished specific structural stability from noise. In contrast, global repetition maps remained highly robust against local noise variations and effectively emphasised the recurrence of structures over time. The global repetition maps combined the evaluation of structure displacement over time with measurements of structure repetition, providing a superior platform for analysis. In these maps, NRMSE values highlighted regions with low NRMSE, indicating that the underlying structures (MTOCs), were significantly more stable compared to EB3 comets. This superior capability of global repetition maps to detect differences between time intervals is attributed to their resistance to noise corruption and their accurate representation of spatiotemporal information. While the average NRMSE values from input images and temporal projections remained stable over time, SReD effectively captured the dynamic nature of microtubules, showcasing its efficacy in tracking and analysing microtubule dynamics (Supplementary Fig. 15b).

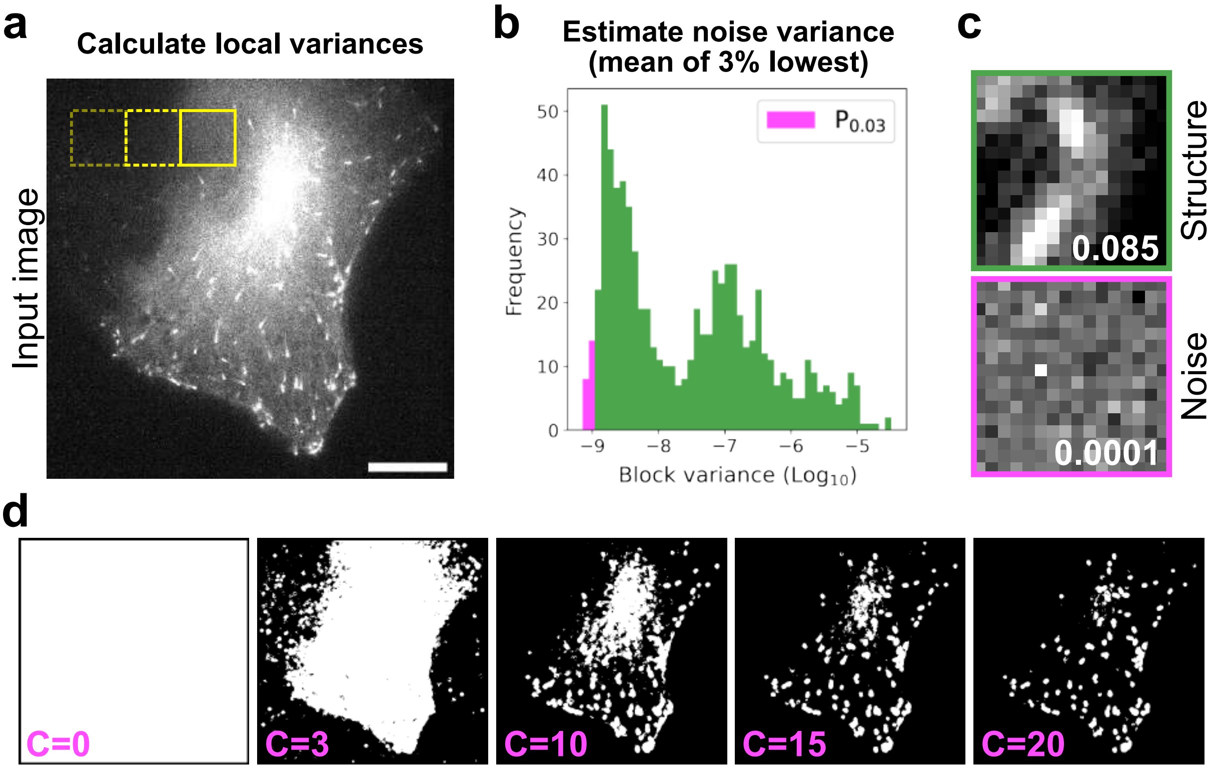

Supplementary Fig. 1. Generation of the relevance mask. a, Representative input image with local variance calculated using non-overlapping blocks, illustrating spatial distribution of image texture. Scale bar: 10 µm. **b,** Histogram of calculated block variances, highlighting the lower tail (magenta, below percentile 0.03) and upper tail (green, above percentile 0.03) of the distribution. Source data are provided as a Source Data file. **c,** Example blocks extracted from the distribution tails: upper tail (green border) showing regions with significant structural information, and lower tail (magenta border) representing predominantly noise. The variance of each block is shown in white text. **d,** Series of relevance masks generated using different relevance constants (denoted as "C"). Each mask is created by multiplying C with the previously calculated average noise variance to determine the final threshold for structural relevance. This demonstrates how adjusting C impacts the discrimination between structurally relevant and irrelevant image regions.

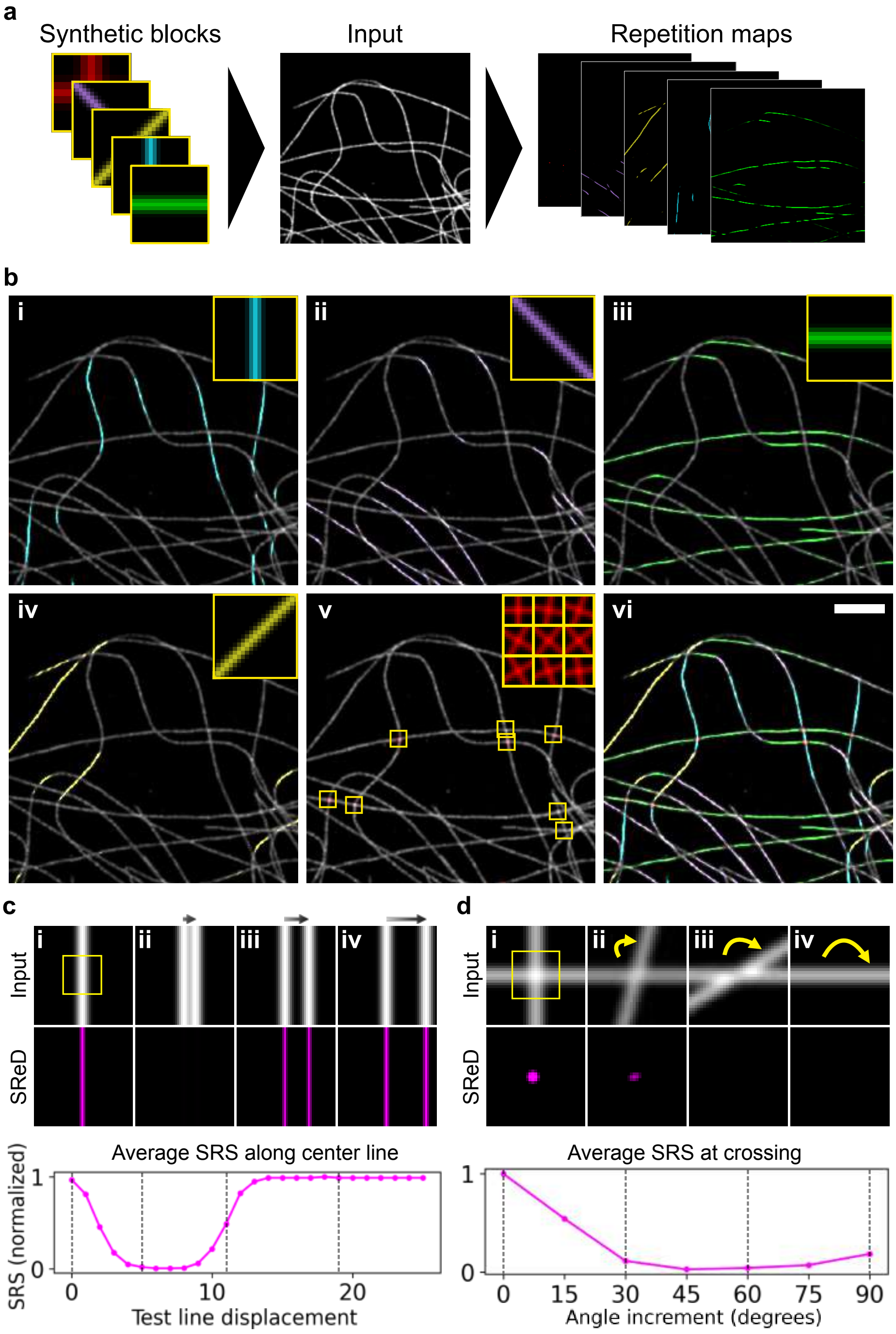

Supplementary Fig. 2. Structure detection using simulated blocks. a, Workflow diagram illustrating the use of simulated reference blocks for structure detection. Simulated blocks containing lines and line crossings at various orientations were generated and used by SReD to analyse a STORM image of microtubules in a HeLa cell. SReD produces a repetition map for each reference block, highlighting regions of structural similarity. **b,** Composite images showing the original STORM reconstruction (grey) overlaid with repetition maps (colour) for each simulated reference block. Scale bar: 2 µm. **c,** Analysis of SReD's specificity for linear structures at varying proximities. (i-iv) Repetition maps generated using a vertical line reference block (yellow box in i) on test images with vertical lines at increasing distances. Graph shows the average Structural Repetition Score (SRS) versus line displacement, with dashed lines corresponding to examples i-iv. **d,** Evaluation of SReD's specificity for complex structures. (i-iv) Repetition maps generated using an orthogonal line crossing reference block (yellow box in i) on test images with line crossings at increasing angles. Graph displays the average SRS versus angle increment, with dashed lines corresponding to examples i-iv. Source data are provided as a Source Data file.

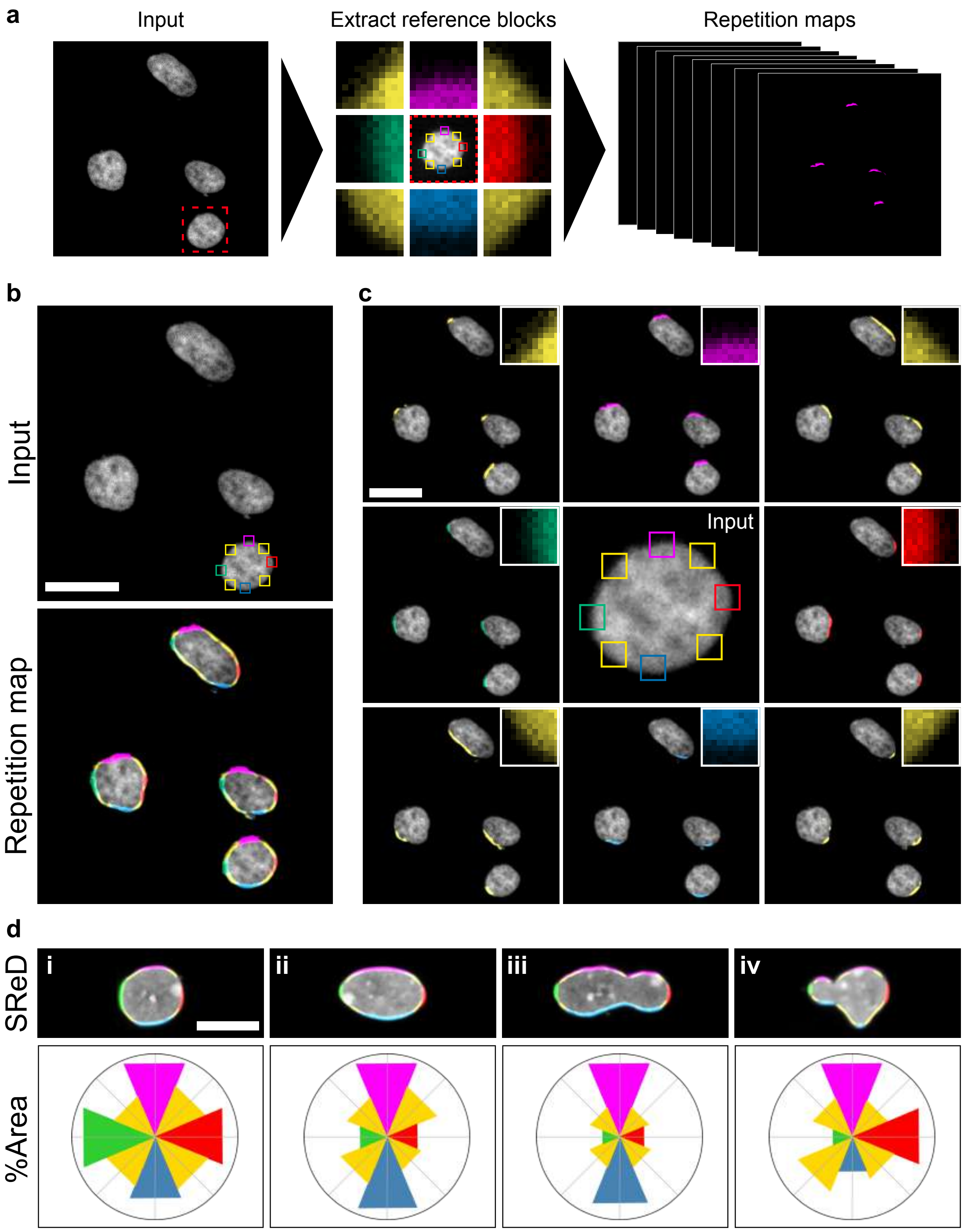

Supplementary Fig. 3. Structure detection using empirical blocks. a, Workflow diagram illustrating the use of empirically extracted reference blocks for structure detection. Reference blocks containing nuclear envelope regions at various orientations were extracted from a DAPI-stained image of HeLa cell nuclei. SReD generates a repetition map for each reference block, highlighting regions of structural similarity. **b,** Top panel: Input image of DAPI-stained HeLa cell nuclei with locations of empirical blocks (i.e., extracted directly from the input data) highlighted. Bottom panel: Composite image showing the overlay of colour-coded repetition maps, each corresponding to a different empirical reference block. Scale bar: 30 µm. **c,** Expanded view of individual repetition maps for each empirical reference block, demonstrating SReD's ability to detect nuclear envelope structures at different orientations. Scale bar: 30 µm. **d,** Top panels: Composite images showing the overlay of colour-coded repetition maps of nuclei i-iv. Scale bar: 30 µm. Bottom panels: Polar plots depicting the relative area percentages of each repetition map in the nuclei composites, demonstrating how SReD can be used to detect different morphological states, possibly related to cell division or stress.

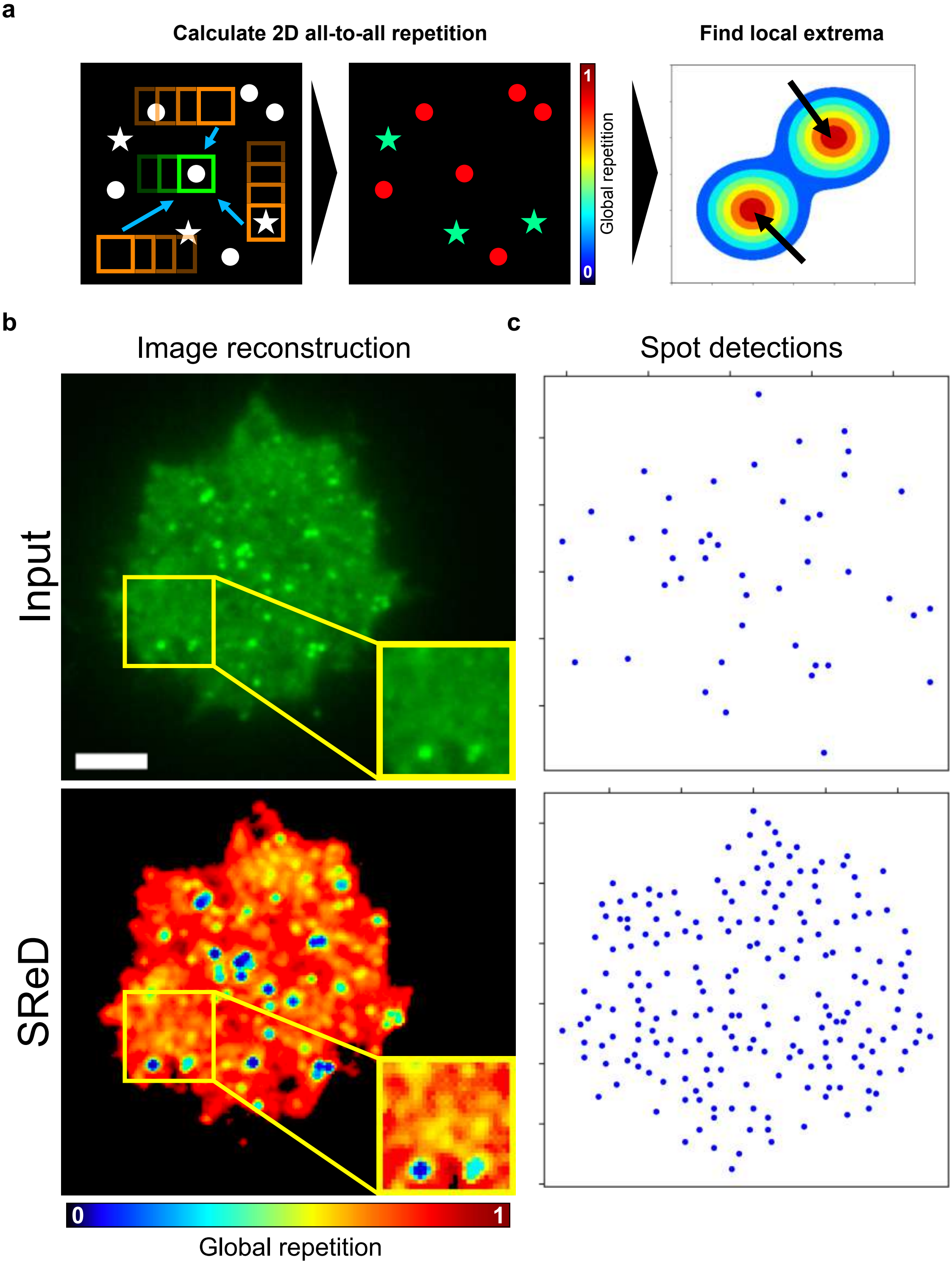

Supplementary Fig. 4. Detection of HIV virus-like particles using Global Repetition. a, Workflow diagram illustrating the "all-to-all" sampling scheme in Global Repetition. The global repetition map is a platform for quantitative analysis such as local extrema calculation. **b,** Top panel: Image reconstruction of an acivated Jurkat cell expressing an HIV Gag-EGFP construct, which induces the assembly of virus-like particles (VLPs). Scale bar: 5 µm. Bottom panel: Global repetition map calculated from the input image in the top panel, using a block size of 7x7 pixels and the "absolute difference of standard deviations" metric. The insets in both panels highlight a region where assembling viral structures can be discerned in the global repetition map but not in the input image. **c,** Local maxima calculated from the input image and the (inverted) global repetition map, showing a higher number of detections in the global repetition map using the same prominence threshold.

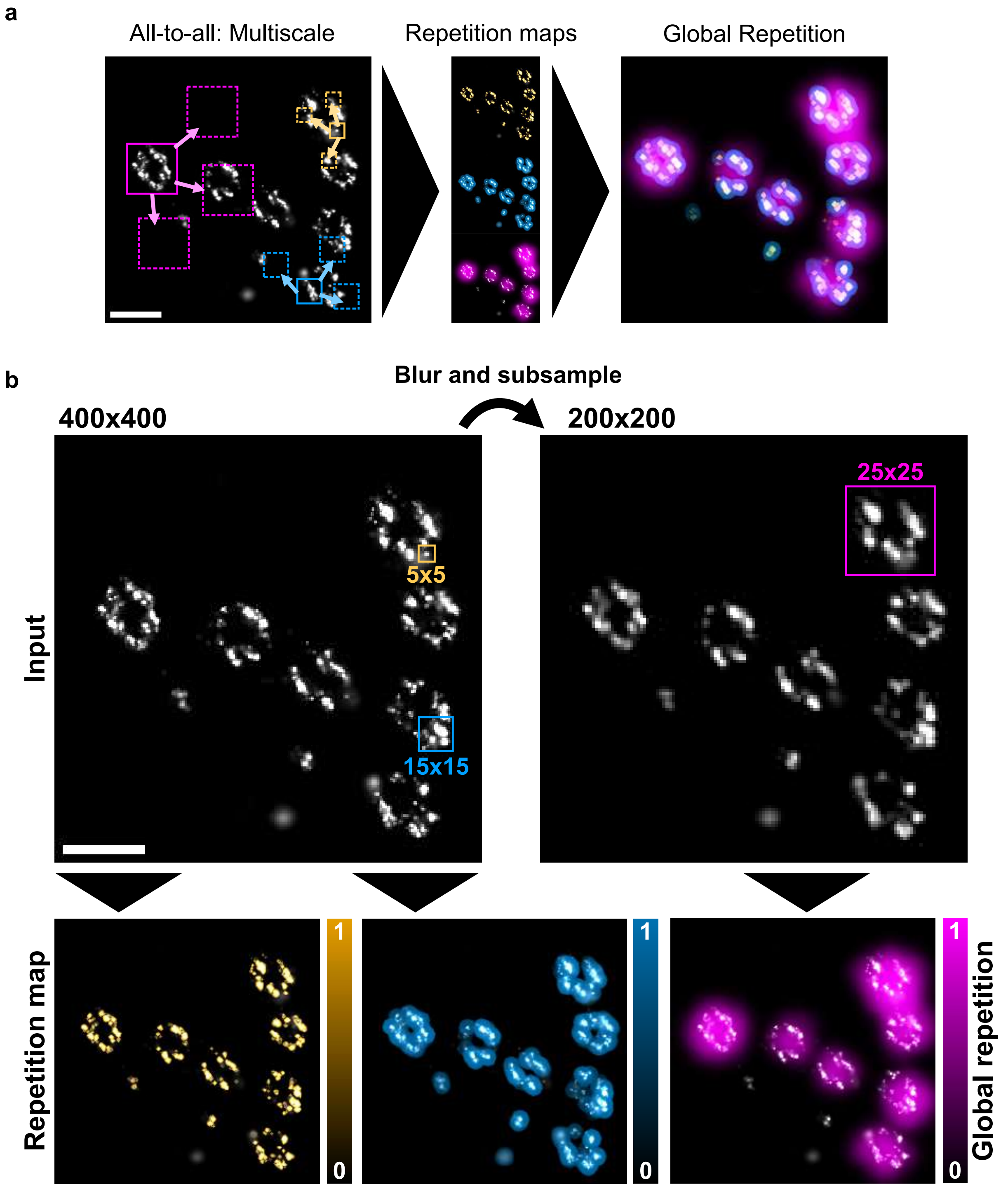

Supplementary Fig. 5. Multiscale detection of Nuclear Pore Complex structures using Global Repetition. a, Workflow diagram illustrating the modulation of block-to-image size ratio modulation for multiscale global repetition analysis. **b,** Image reconstructions of the input image and the block sizes used for global repetition analysis. A low- and intermediate- order analysis used the original image dimensions (400x400 pixels) and 5x5 and 15x15 pixel block sizes to detect single nucleoporins (orange) and nucleoporin clusters (blue). A high-order analysis used a downscaled input image (200x200 pixels) and a block size of 25x25 pixels to detect entire NPC units.

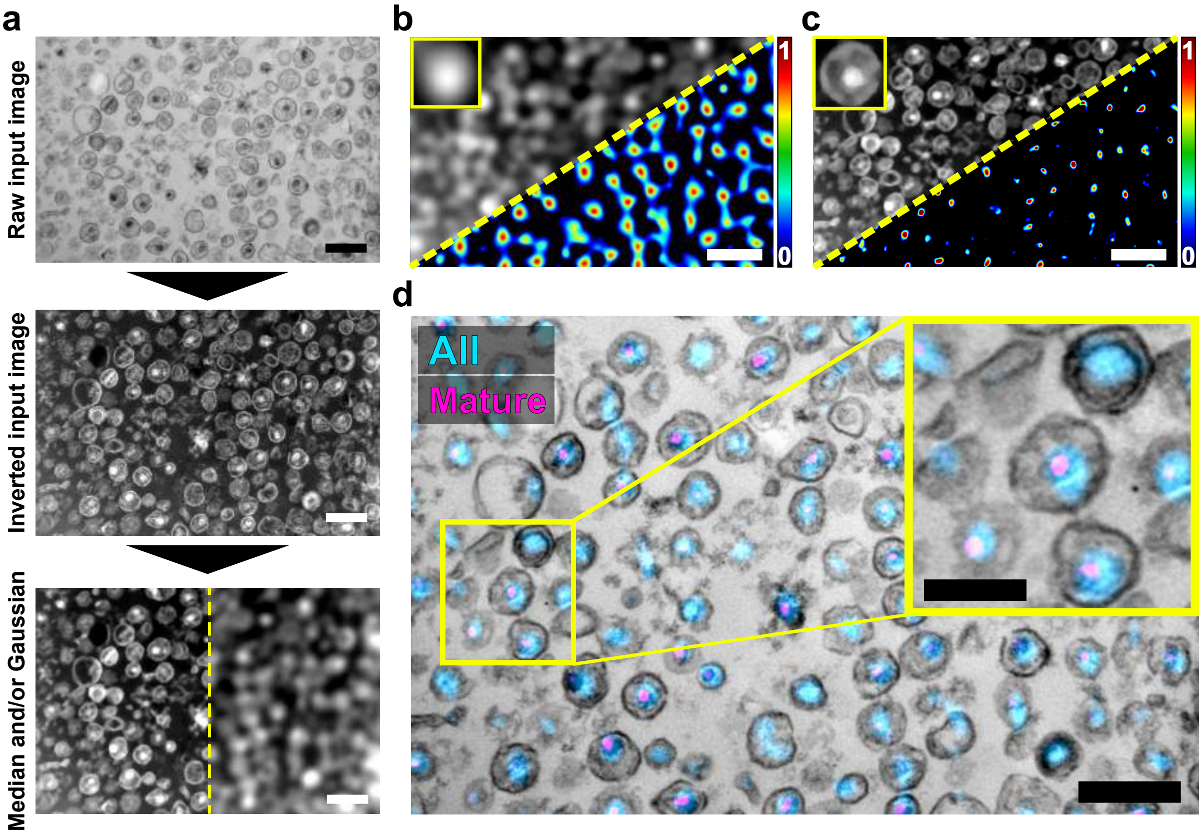

Supplementary Fig. 6. Detection of HIV particles in transmission electron microscopy data. a, Schematic of the data preprocessing tasks preformed. The raw input image was first inverted and filtered according to the specific structures being analysed: To analyse particles containing mature capsids, a Median filter with a radius of 5 pixels was applied; for particles of all types, an additional blurring step was performed using a 10-pixel Gaussian kernel**. b,** An empirical reference block (yellow square) representing a generic particle was extracted from the Gaussian-blurred input image (top left half) and used for block repetition analysis. The block repetition map (bottom right half) highlights repetitions of the reference block. **c,** An empirical reference block (yellow square) representing a particle containing a mature capsid was extracted from the Median-filtered input image (top left half) and used for block repetition analysis. The block repetition map (bottom right half) highlights repetitions of the reference block. **d,** Overlay of the raw input image and the block repetition maps calculated in panels b) and c), highlighting regions where generic particles ("All", cyan) and particles containing mature capsids ("Mature", magenta) can be found. Scale bars: 300 nm (main panels), 150 nm (inset).

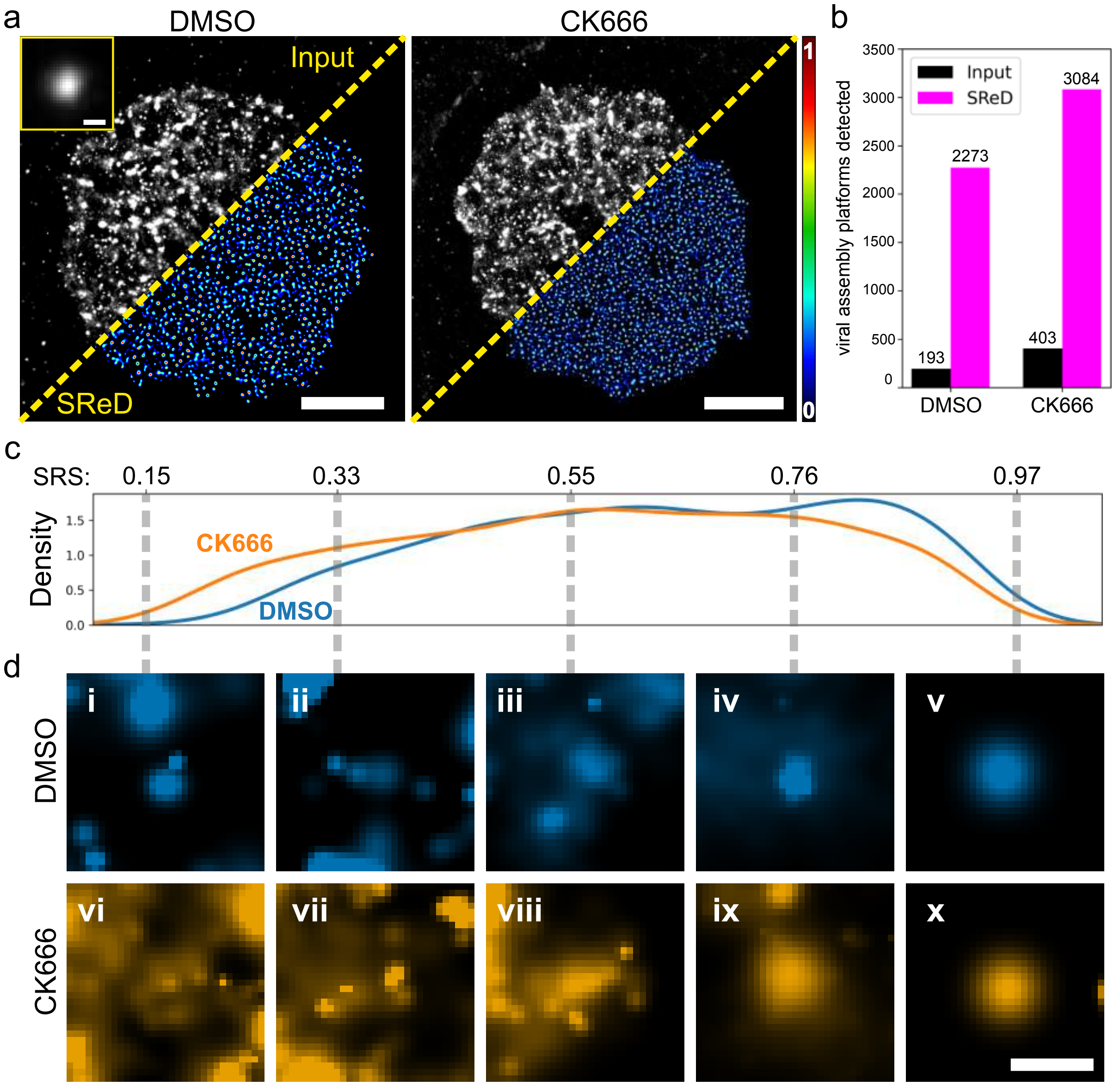

Supplementary Fig. 7. Detection and quantitative analysis of HIV assembly platforms in STORM data. a, Input image reconstructions (top left halves) and SReD repetition maps (bottom right halves) calculated using an empirical reference block extracted from the control (DMSO) image (inset). Scale bars: 3 µm (main panels), 100 nm (inset). **b,** Bar plot showing the number of viral assembly platforms detected, which was higher in the CK666 groups compared to the corresponding DMSO, irrespective of the method (direct input analysis (black) vs. SReD repetition maps (magenta)). A higher number of platforms were detected in the SReD repetition maps compared to direct input analysis. **c,** Density histogram showing the SRS distributions of the viral assembly platforms detected in the SReD repetition maps. Lines correspond to kernel density estimates of the original histogram. **d,** Representative examples of the viral assembly platforms detected (i-x). Scale bar: 100 nm. Source data are provided as a Source Data file.

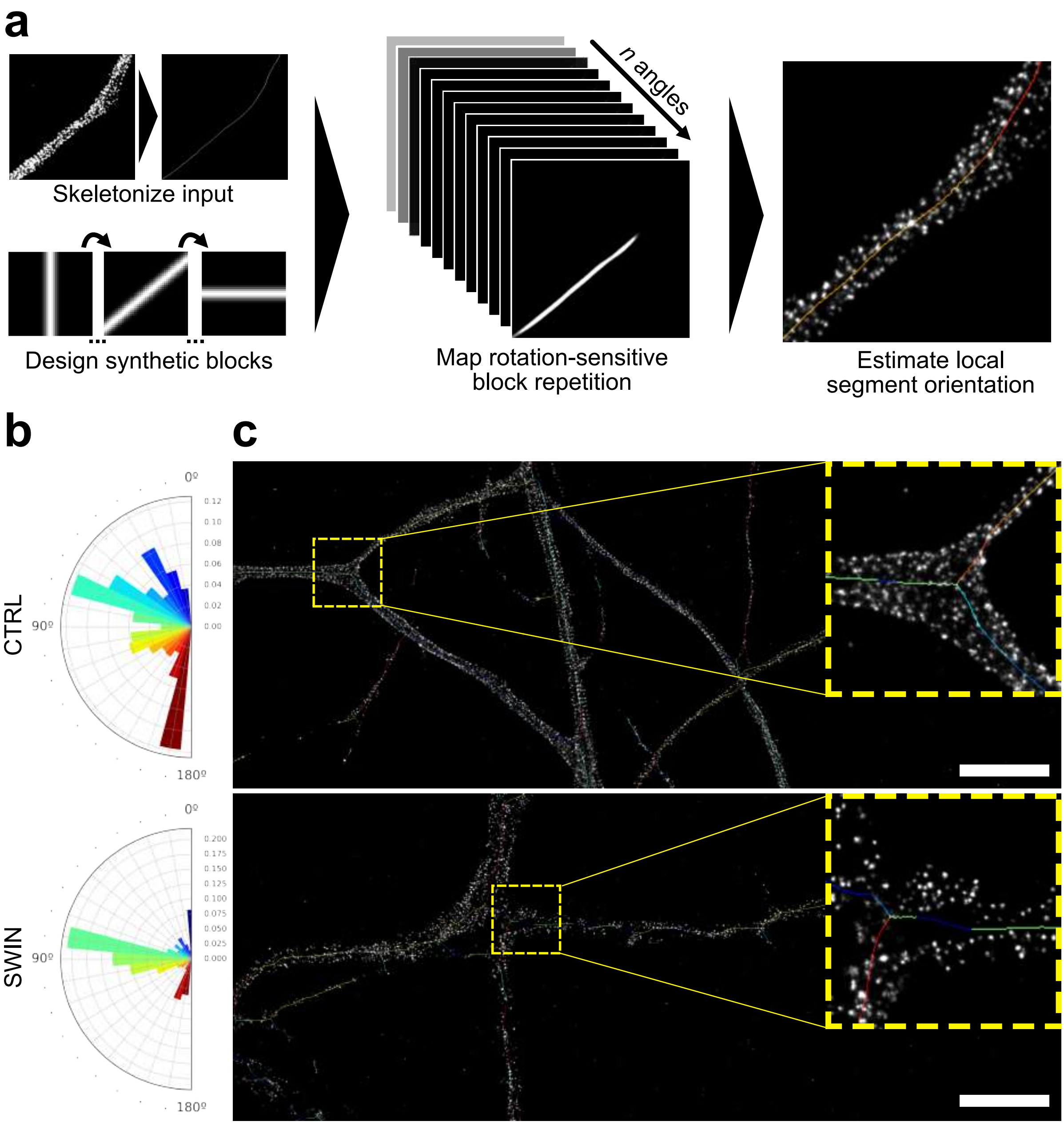

Supplementary Fig. 8. Automated estimation of axon orientations. a, Workflow diagram illustrating the estimation of axon orientations. Synthetic blocks comprising lines at different orientations are used to calculate repetitions in skeletonised axons. Each skeleton coordinate is labelled with the angle corresponding to the highest SRS. **b,** Polar plots depicting the distribution of axon angles in the control (top panel) and swinholide A (bottom panel) samples. **c,** Overlay of representative input images and their angle-labelled skeletons, using the same colour code used in panel b). Scale bar: 5 µm. Source data are provided as a Source Data file.

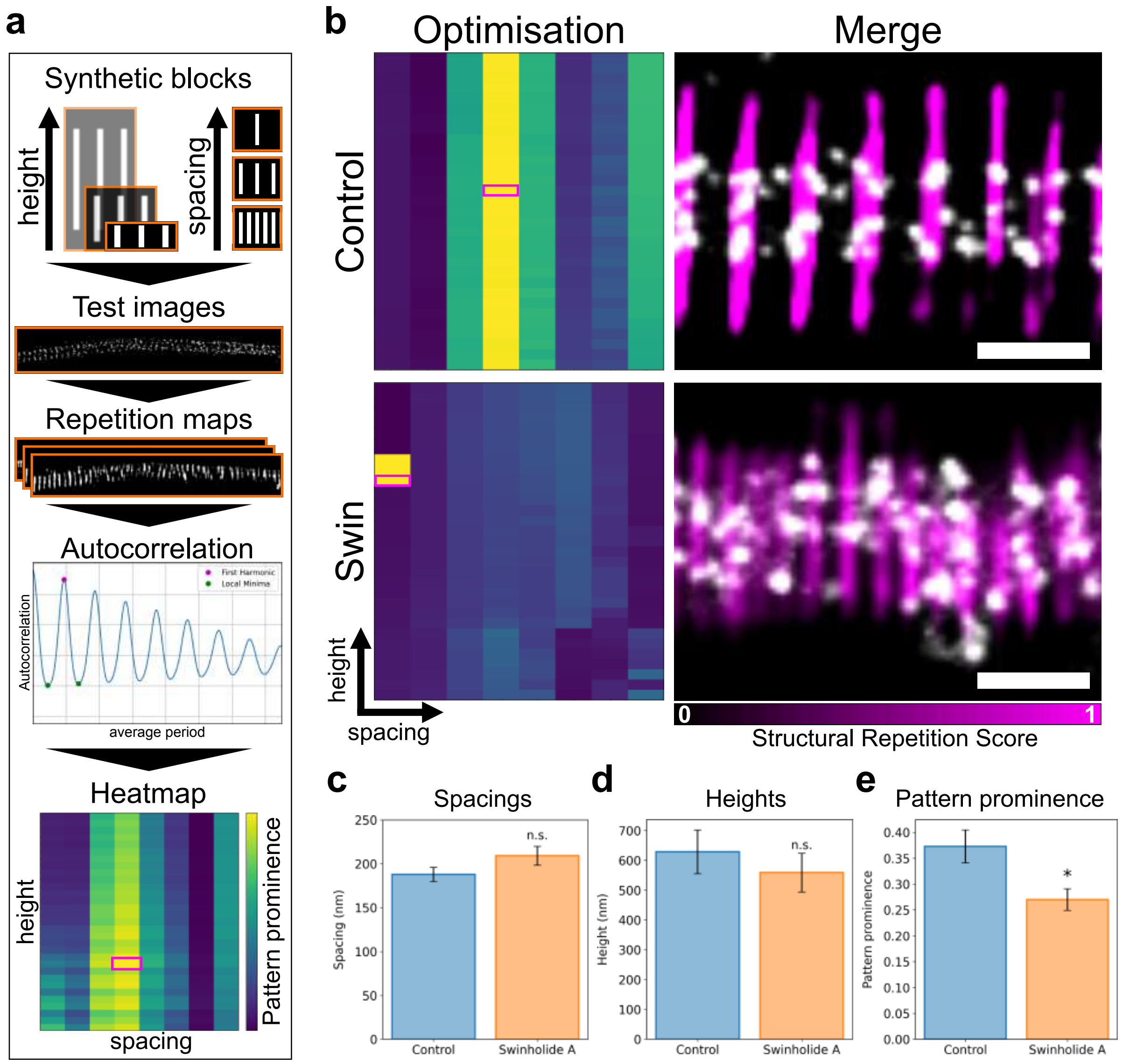

Supplementary Fig. S9. Optimisation of block parameters for ring pattern detection. a, Schematic representation of the tasks performed. A set of 248 synthetic blocks with different spacing and height combinations were generated. Test images (30 for each experimental group) were randomly extracted from each dataset and SReD was used to calculate the repetition maps of each reference block. The repetition maps were analysed using autocorrelation functions and the patterns' prominence (given by the autocorrelations' first harmonic amplitude) was used to assess how well each reference block fitted the data. For each test image, this information is depicted in a heatmap. The combination of parameters with the highest pattern prominence value is chosen from each heatmap and averaged within each experimental group to calculate the optimised block. **b,** Representative examples of the optimisation process. The heatmaps where the highest pattern prominences were found are shown. Scale bar: 0.4 µm. **c-e,** Plots of the optimised parameters calculated by comparing the distributions of each parameter between groups (n=248, n.s. p>0.05, * p<0.05, Mann-Whitney U test).

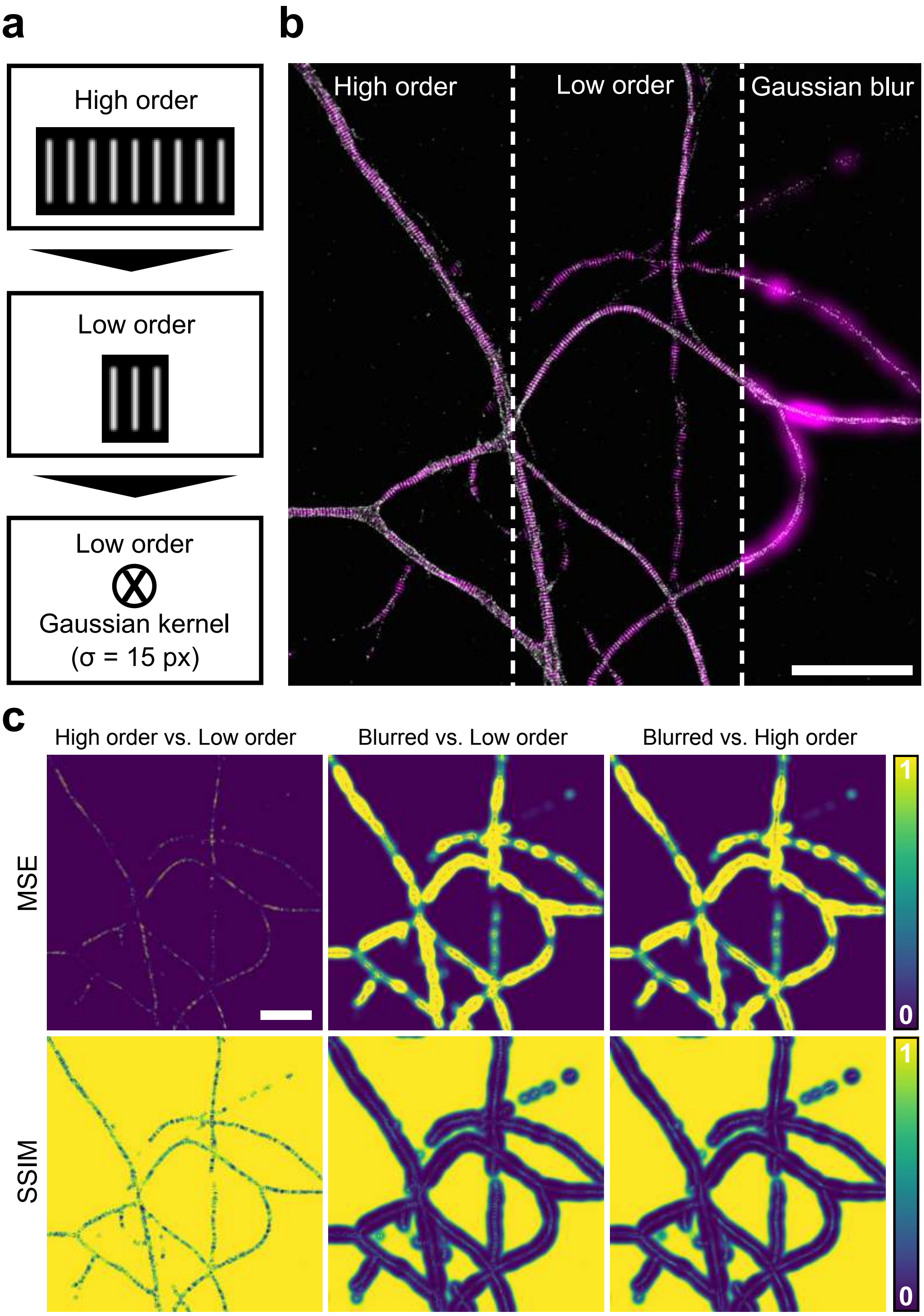

Supplementary Fig. 10. Detection of high-order ring patterns. a, Schematic representation of the samples generated. Top panel: Reference block containing a high-order ring pattern with 9 rings. Center panel: Reference block containing a low-order ring pattern with 3 rings. Bottom panel: Convolution with a Gaussian kernel (15 px radius). **b,** Overlay of a representative example of the input data and the corresponding block repetition maps. **c,** Error maps demonstrating that SReD enables detecting high-order patterns without discarding low-order information. The high-order repetition maps are a more robust representation of the low-order structures compared to the method using convolutions with Gaussian kernels. Scale bars: 5 µm. Source data are provided as a Source Data file.

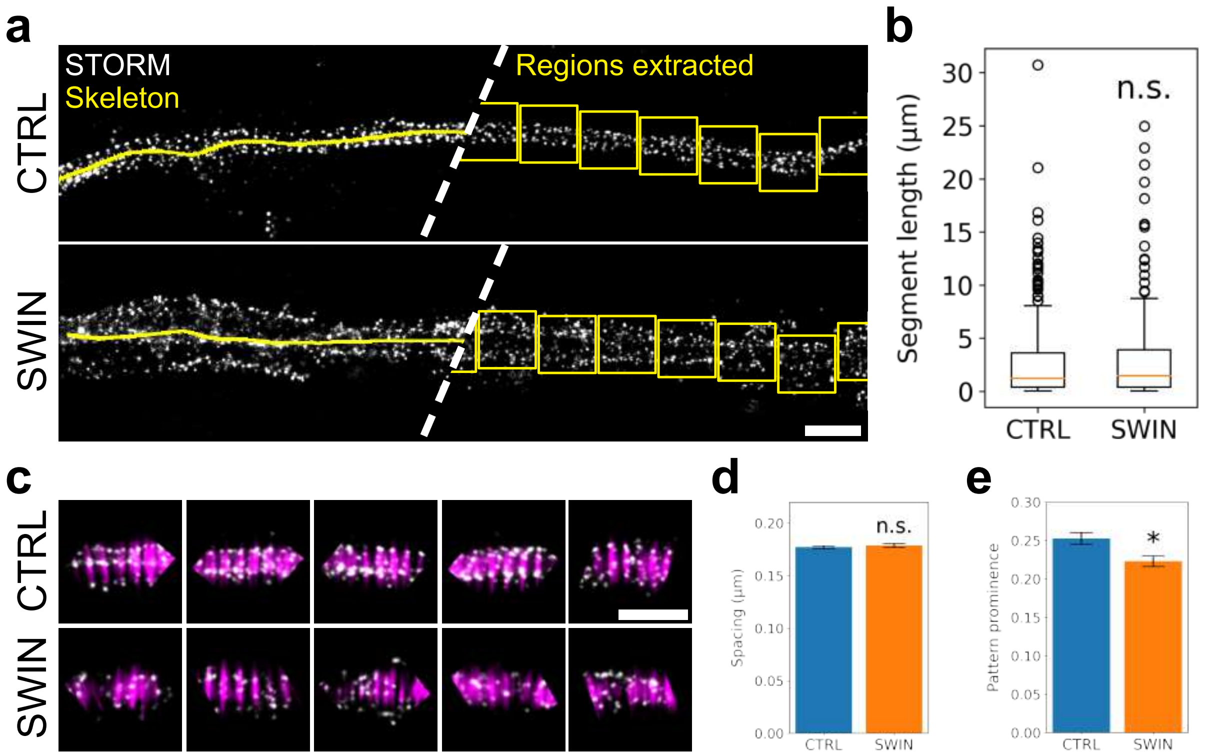

Supplementary Fig. 11. Quantitative analysis of ring patterns in axon segments. a, Image reconstructions illustrating the approach used to automatically extract non-overlapping regions along the axons' skeletons. Scale bar: 1 μm. **b,** Length distributions of the skeletonised axon segments analysed, showing no significant differences between experimental groups. **c,** Representative examples of the non-overlapping regions extracted along the axons' skeleton segments (STORM in gray, SReD repetition maps in magenta). Scale bar: 1 μm. **d,** Average spacing of the regions analysed, showing no significant differences between experimental groups (N=6, mean ± SEM - CTRL: 177 nm ± 1 nm, SWIN: 179 nm ± 2 nm, n.s. p>0.05, t-test). **e,** Pattern prominence of the regions analysed, showing that swinholide A (SWIN) treatment resulted in a significant decrease of the average pattern prominence (N=6, mean ± SEM - CTRL: 0.253 ± 0.008, SWIN: 0.223 ± 0.007, * p<0.05, t-test). Source data are provided as a Source Data file.

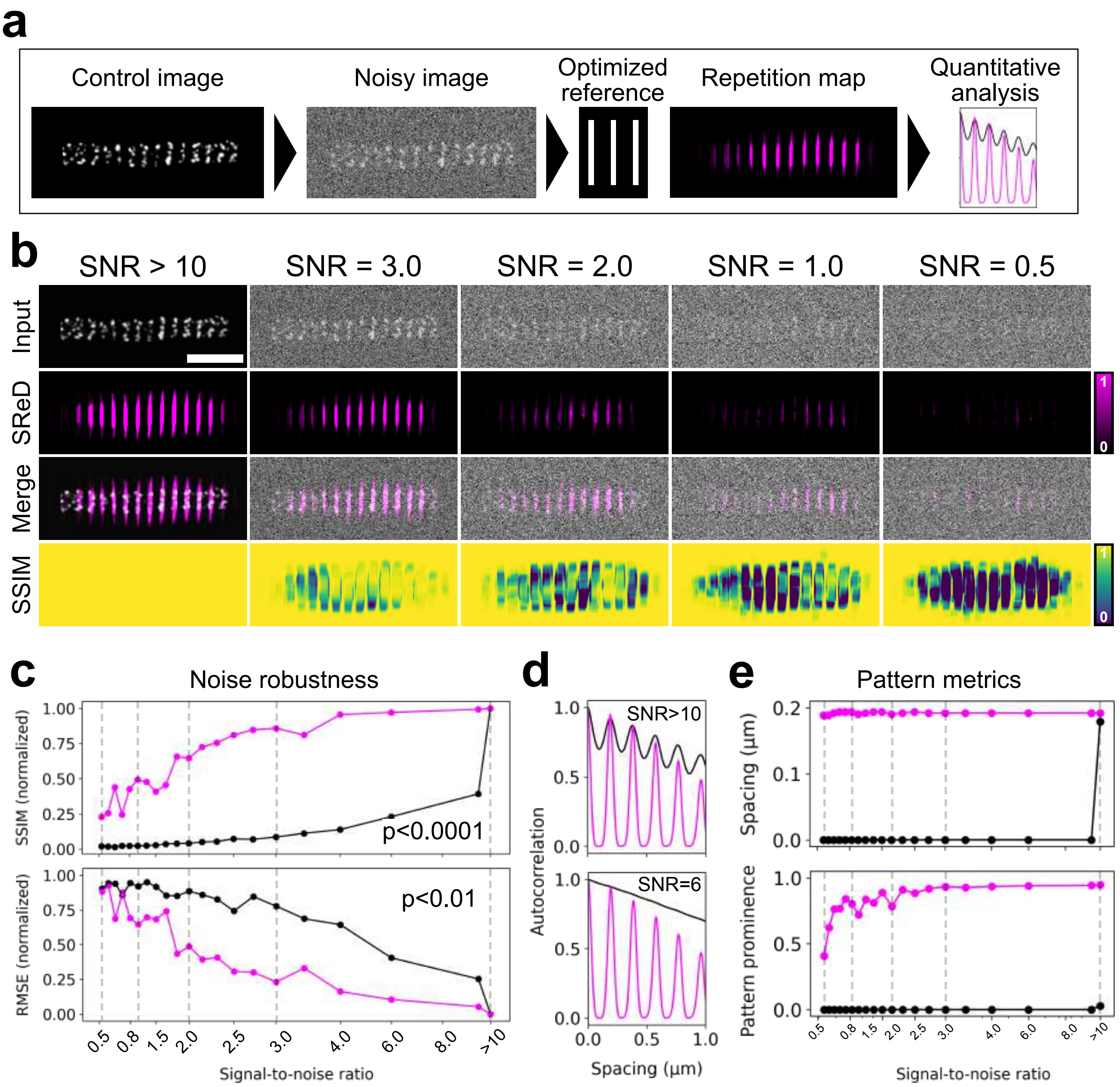

Supplementary Fig. 12. Evaluation of SReD's performance in images containing random noise. **a,** Schematic of the tasks performed. A control image containing a periodic pattern is corrupted with random noise. The periodic pattern is mapped using an optimised reference block. The repetition maps are quantitatively analysed using autocorrelation functions. **b,** Visual comparison of image reconstructions at different signal-to-noise ratios (SNRs). Top row: Input images with SNRs decreasing from left to right. Middle row: Repetition maps calculated using an optimised reference block. Bottom row: Merge of the input images and the repetition maps. Scale bar: 1 µm. **c,** Evaluation of noise robustness by comparing the distributions of control vs. noisy images (black) and the corresponding repetition maps (magenta) at different SNRs using different metrics. The repetition maps provided a superior representation of the control conditions in all cases. Top panel: Distributions calculated using the Structural Similarity Index (SSIM) metric ($p<0.0001$, t-test). Bottom panel: Distributions calculated using the Root Mean Squared Error (RMSE) metric ($p<0.01$, t-test). **d,** Autocorrelation plots demonstrating the sensitivity to the periodic patterns in the input images (black) and the SReD repetition maps (magenta). **e,** Distribution of the inter-ring spacing (top panel) and pattern prominence (bottom panel) calculated from the autocorrelation functions of the input data (black) and the SReD repetition maps (magenta) at different SNRs. Source data are provided as a Source Data file.

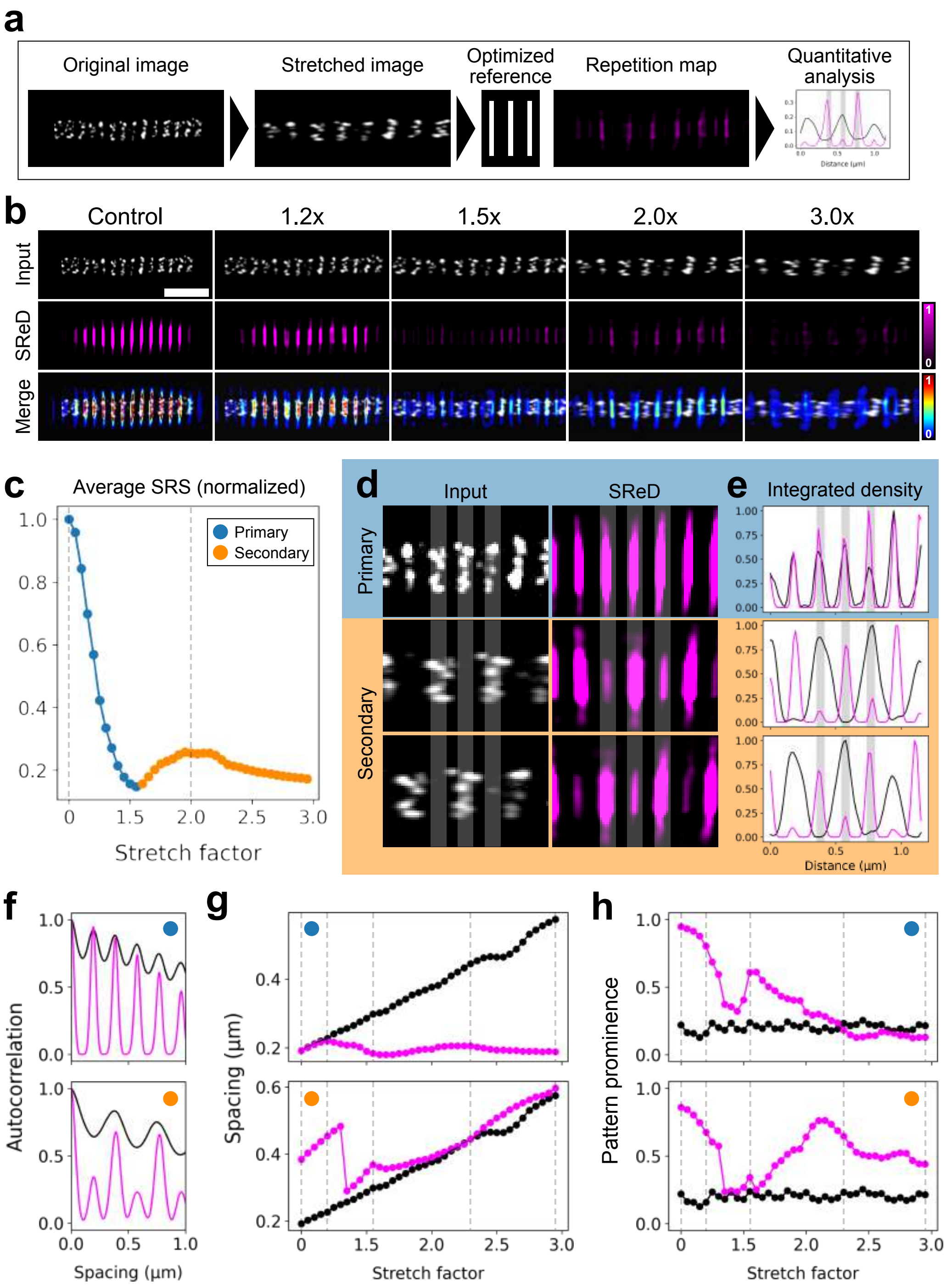

Supplementary Fig. 13. Evaluation of SReD's specificity. a, Schematic of the tasks performed. A control image containing a periodic pattern is stretched along its width to disrupt the pattern's characteristics. The periodic pattern is mapped using an optimised reference block. The repetition maps are quantitatively analysed using autocorrelation functions. **b,** Visual comparison of image reconstructions at different stretch factors. Top row: Input images with stretch factors increasing from left to right. Middle row: Repetition maps calculated using an optimised reference block. Bottom row: Merge of the input images and the repetition maps. Scale bar: 1 µm. **c,** Average Structural Repetition Factor (SRS) in the repetition maps plotted against the stretch factor. A primary pattern (blue) and a secondary pattern (orange) are detected. **d,** Image reconstructions of the primary and secondary patterns detected in c). **e,** 2D intensity profiles (i.e., integrated density) of the images in d). **f,** Autocorrelation plots of the input images (black) and repetition maps (magenta) at stretch factors 0 (top) and 2.0 (bottom). **g,** Inter-ring spacings calculated from the autocorrelation analysis plotted against the stretch factor. **h,** Pattern prominences calculated from the autocorrelation analysis plotted against the stretch factor. The dashed gray lines indicate the samples shown in panel b).

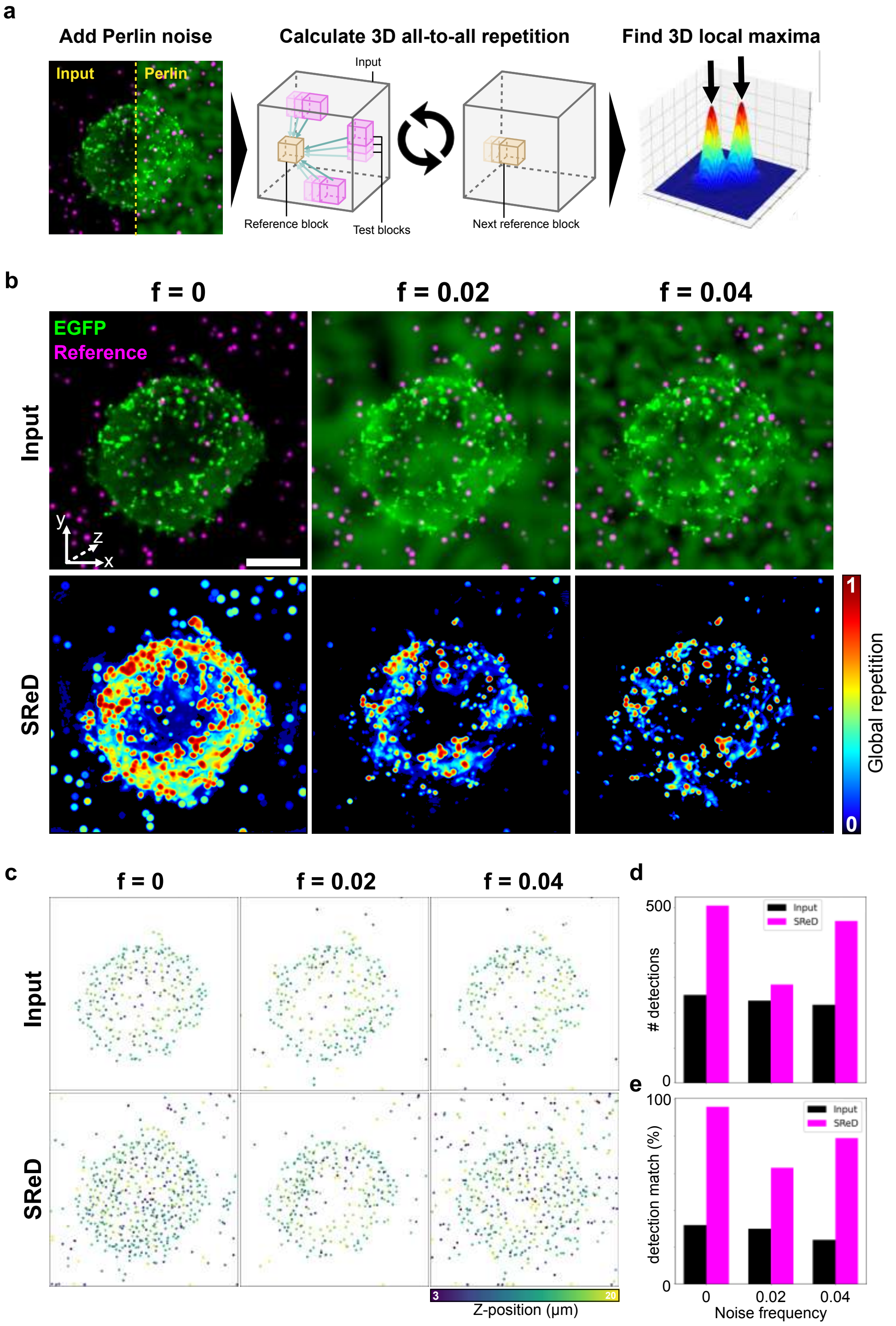

Supplementary Fig. 14. Evaluation of SReD's performance in images corrupted with non-specific structures. a, Schematic of the tasks performed. Perlin noise is added to a 3D image containing a Jurkat cell expressing an HIV Gag-EGFP construct (green) and synthetic bead structures resembling free viral particles (magenta). A Global Repetition map is calculated and 3D local maxima are calculated. **b,** Input images with Perlin noise of different frequencies and their corresponding Global Repetition maps. **c,** Plots of the spot detections obtained by calculating local maxima in the input images and their Global Repetition maps. **d,** Plot showing the number of detections obtained in each sample. e, Plot showing the percentage of reference detections obtained in each sample.

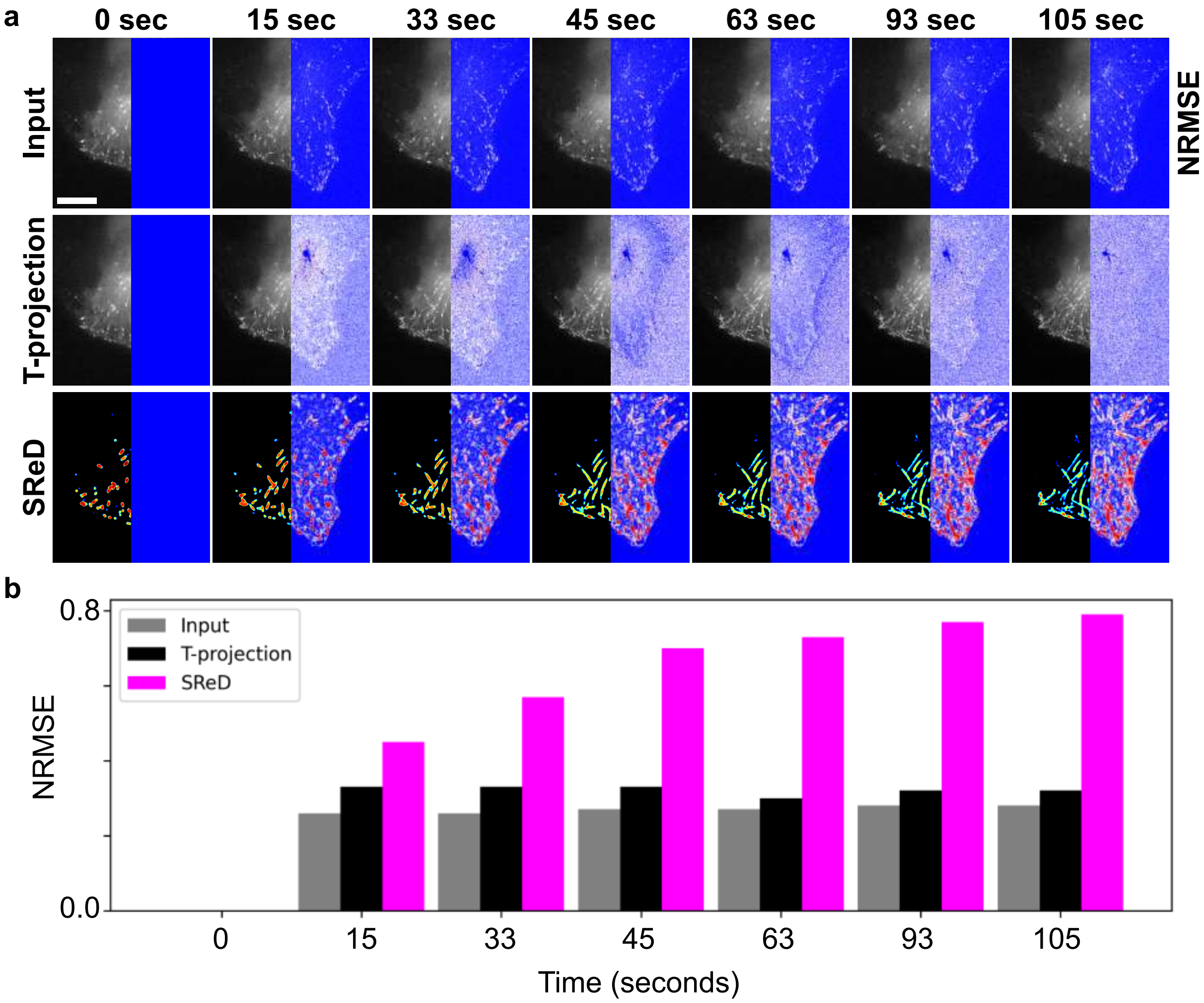

Supplementary Fig. 15. Assessment of the microtubule network's stability along time using Global Repetition. a, Analysis of structural stability by comparing different time frames with the initial state (0 sec) using the Normalised Root Mean Squared Error (NRMSE) metric. Top row: Input images showing the final state in each time frame. Middle row: Time projections of all time points within each time frame. Bottom row: SReD global repetition maps calculated using time as the third dimension of the analysis. **b,** Plot showing the NRMSE distributions of each dataset.

Supplementary Table 1. Comparative analysis of SReD's functionalities against other methods in the same domain.

Method	Approach	Example	Scaling Robustness	Rotation Robustness	No Structural Priors Required	GUI	Implementation	Input Type	Output type	Applications
Programatic template matching[14]	Comparison of predefined templates with the data using normalized cross-correlations	Scikit Template Matching	✗	✗	✗	✗	Python	Multimodal image data	Similarity map	General image analysis, quality control, object recognition
Cryo-EM template matching	High-resolution template matching in cryo-EM data to localize macromolecular complexes	STOPGAP[15]	✗	✓	✗	✗	MATLAB	Subtomogram data (e.g., tilt series)	3D structural models	Macromolecular structure determination
Feature-based detection	Extracting keypoints and descriptors from images	SIFT[16], *SURF*[17]	✓	✓	✓	✗	C++[16], *Python*[17]	Multimodal image data	Keypoints and descriptors	Object recognition, image stitching, real-time tracking
Deep-learning-based detection	Using CNNs to learn features and detect patterns in images	U-Net[18]	✓	✓	✗	✗	Python	Multimodal image data	Various (e.g., bounding boxes, segmentation masks)	Wide range of computer vision tasks
Point-based detection	Using single molecule detections for coordinate-oriented template matching	SPARTAN[19], *smlm_datafusion2D*[20]	✓	✓	✗	✓	MATLAB	Point data	Coordinate-based 2D/3D models	Particle detection and registration
SReD (ours)	Calculating quantitative maps of structural repetition using correlation metrics	-	✓	✓	✓	✓	ImageJ/Fiji	Multimodal image data	Structural repetition maps	Biological microscopy, extendable to all image analysis

Supplementary Bibliography

1. Jérôme Boulanger, Charles Kervrann, Patrick Bouthemy, Peter Elbau, Jean-Baptiste Sibarita, and Jean Salamero. Patch-Based Nonlocal Functional for Denoising Fluorescence Microscopy Image Sequences. IEEE Transactions on Medical Imaging, 29(2):442–454, February 2010. ISSN 1558-254X. doi: 10.1109/TMI.2009.2033991. Conference Name: IEEE Transactions on Medical Imaging.
2. Wenjiang Liu, Tao Liu, Mengtian Rong, Ruolin Wang, and Hao Zhang. A fast noise variance estimation algorithm. In 2011 Asia Pacific Conference on Postgraduate Research in Microelectronics & Electronics, pages 61–64, October 2011. doi: 10.1109/PrimeAsia.2011.6075071. ISSN: 2159-2160.
3. Angélique Jimenez, Karoline Friedl, and Christophe Leterrier. About samples, giving examples: Optimized Single Molecule Localization Microscopy. Methods, 174:100–114, March 2020. ISSN 1046-2023. doi: 10.1016/j.ymeth.2019.05.008.
4. Olivier Burri and Romain Guiet. DAPI and Phase Contrast Images Dataset, May 2019.
5. Juan S. Rey, Wen Li, Alexader J. Bryer, Hagan Beatson, Christian Lantz, Alan N. Engelman, Juan R. Perilla. Deep-learning in situ classification of HIV-1 virion morphology. Computational and Structural Biotechnology Journal, 19:5688-5700, October 2021. doi: 10.1016/j.csbj.2021.10.001.
6. Nobuyuki Otsu. A Threshold Selection Method from Gray-Level Histograms. IEEE Transactions on Systems, Man, and Cybernetics, 9(1):62–66, January 1979. ISSN 2168-2909. doi: 10.1109/TSMC. 1979.4310076. Conference Name: IEEE Transactions on Systems, Man, and Cybernetics.
7. Rayane Dibsy, Erwan Bremaud, Johnson Mak, Cyril Favard & Delphine Muriaux. Hiv-1 diverts cortical actin for particle assembly and release. Nature Communications. 14, 6945 (2023). doi: 10.1038/s41467-023-41940-0.
8. Martin Ovesný, Pavel Křížek, Josef Borkovec, Zdeněk Švindrych, Guy M Hagen. ThunderSTORM: a comprehensive ImageJ plug-in for PALM and STORM data analysis and super-resolution imaging. Bioinformatics. 2014 Apr 25;30(16):2389-2390. doi: 10.1093/bioinformatics/btu202.
9. Tao Huang, Heng Peng and Kun Zhang. Model selection for Gaussian mixture models. Statistica Sinica, 27(1):147-169 (2017). doi: 10.5705/ss.2014.105.
10. Akaike, H. "A new look at the statistical model identification." IEEE Transactions on Automatic Control, 19(6), 716–723 (1974). doi: 10.1109/TAC.1974.1100705.
11. Schwarz, G. Estimating the dimension of a model. The Annals of Statistics, 6(2), 461–464 (1978). doi: 10.1214/aos/1176344136
12. E. Meijering, M. Jacob, J.-C.f. Sarria, P. Steiner, H. Hirling, and M. Unser. Design and validation of a tool for neurite tracing and analysis in fluorescence microscopy images. Cytometry Part A, 58A(2):167–176, 2004. ISSN 1552-4930. doi: 10.1002/cyto.a.20022._eprint: https://onlinelibrary.wiley.com/doi/pdf/10.1002/cyto.a.20022.
13. Stéphane Vassilopoulos, Solène Gibaud, Angélique Jimenez, Ghislaine Caillol, and Christophe Leterrier. Ultrastructure of the axonal periodic scaffold reveals a braid-like organization of actin rings. Nature Communications, 10(1):5803, December 2019. ISSN 2041-1723. doi: 10.1038/s41467-019-13835-6.
14. Nazanin Sadat Hashemi, Roya Babaie Aghdam, Atieh Sadat Bayat Ghiasi, Parastoo Fatemi. Template matching advances and applications in image analysis. Arxiv (2016). doi: 10.48550/arXiv.1610.07231.
15. Sergio Cruz-León, Tomáš Majtner, Patrick C. Hoffmann, Jan Philipp Kreysing, Sebastian Kehl, Maarten W. Tuijtel, Stefan L. Schaefer, Katharina Geißler, Martin Beck, Beata Turoňová, Gerhard Hummer. High-confidence 3D template matching for cryo-electron tomography. Nature Communications 15, 3992 (2024). doi: 10.1038/s41467-024-47839-8.
16. D.G. Lowe. Object recognition from local scale-invariant features. IEEE Xplore. Proceedings of the Seventh IEEE International Conference on Computer Vision. 2'-27 September 1999. doi: 10.1109/ICCV.1999.790410.
17. Herbert Bay, Andreas Ess, Tinne Tuytelaars and Luc Van Gool. Speeded-up robust features (SURF). Computer Vision and Image Understanding. Vol. 110(3):346-359 (2008). doi: 10.1016/j.cviu.2007.09.014.
18. Oaf Ronneberger, Philipp Fischer, Thomas Brox. U-Net: convolutional networks for biomedical image segmentation. ArXiv (2015). doi: 10.48550/arXiv.1505.04597.
19. Christian Sieben, Niccolò Banterle, Kyle M. Douglass, Pierre Gonczy, Suliana Manley. Multicolor single-particle reconstruction of protein complexes. Nature Methods. 15:777-780 (2018). doi: 10.1038/s41592-018-0140-x.

20. Hamidreza Heydarian, Florian Schueder, Maximilian T. Strauss, Ben van Werkhoven, Mohamadreza Fazel, Keith A. Lidke, Ralf Jungmann, Sjoerd Stallinga, Bernd Rieger. Template-free 2D particle fusion in localization microscopy. Nature Methods. 15:781-784 (2018). doi: 10.1038/s441592-018-0136-6.
