# Supplementary material for: Structural Repetition Detector for multi-scale quantitative mapping of molecular complexes through microscopy
Source: Nat Commun. 2025 Jul 1;16:5767. doi: 10.1038/s41467-025-60709-1 (PMC12219329; doi:10.1038/s41467-025-60709-1)
Supplement: Supplementary file 2 — Reporting Summary [file 41467_2025_60709_MOESM2_ESM.pdf]

nature portfolio

Corresponding author(s): Ricardo Henriques

Last updated by author(s): May 13, 2025

Reporting Summary

Nature Portfolio wishes to improve the reproducibility of the work that we publish. This form provides structure for consistency and transparency in reporting. For further information on Nature Portfolio policies, see our Editorial Policies and the Editorial Policy Checklist.

Statistics

For all statistical analyses, confirm that the following items are present in the figure legend, table legend, main text, or Methods section.

n/a	Confirmed	
☐	☒	The exact sample size (n) for each experimental group/condition, given as a discrete number and unit of measurement
☐	☒	A statement on whether measurements were taken from distinct samples or whether the same sample was measured repeatedly
☐	☒	The statistical test(s) used AND whether they are one- or two-sided<br>*Only common tests should be described solely by name; describe more complex techniques in the Methods section.*
☐	☒	A description of all covariates tested
☐	☒	A description of any assumptions or corrections, such as tests of normality and adjustment for multiple comparisons
☐	☒	A full description of the statistical parameters including central tendency (e.g. means) or other basic estimates (e.g. regression coefficient) AND variation (e.g. standard deviation) or associated estimates of uncertainty (e.g. confidence intervals)
☐	☒	For null hypothesis testing, the test statistic (e.g. F, t, r) with confidence intervals, effect sizes, degrees of freedom and P value noted<br>*Give P values as exact values whenever suitable.*
☒	☐	For Bayesian analysis, information on the choice of priors and Markov chain Monte Carlo settings
☒	☐	For hierarchical and complex designs, identification of the appropriate level for tests and full reporting of outcomes
☐	☒	Estimates of effect sizes (e.g. Cohen's d, Pearson's r), indicating how they were calculated

Our web collection on statistics for biologists contains articles on many of the points above.

Software and code

Policy information about availability of computer code

Data collection	No data collection software was used in this study.
Data analysis	The SReD algorithm is an open-source distribution and is available at the SReD Github repository (https://github.com/HenriquesLab/SReD/). All custom scripts used in the manuscript are available in the SReD Github repository (https://github.com/HenriquesLab/SReD/tree/main/Scripts/). Analyses were performed using ImageJ/Fiji (https://imagej.net/software/fiji/) and custom Python scripts.

For manuscripts utilizing custom algorithms or software that are central to the research but not yet described in published literature, software must be made available to editors and reviewers. We strongly encourage code deposition in a community repository (e.g. GitHub). See the Nature Portfolio guidelines for submitting code & software for further information.

Data

Policy information about availability of data

All manuscripts must include a data availability statement. This statement should provide the following information, where applicable:

- Accession codes, unique identifiers, or web links for publicly available datasets
- A description of any restrictions on data availability
- For clinical datasets or third party data, please ensure that the statement adheres to our policy

The data obtained in this study is available at https://doi.org/10.5281/zenodo.13764726 and https://doi.org/10.6019/S-BIAD1620 under CC BY 4.0 license.
The STORM data containing cells with labelled microtubules is available at https://doi.org/10.5281/zenodo.5534351 (10). The widefield microscopy data containing

DAPI-stained nuclei is available at https://doi.org/10.5281/zenodo.3232478 (11). The STORM data containing nuclear pores with labelled gp210 is available at https://www.embl.de/download/ries/excitation_intensities/Nup96-BG383/AF647_250kWcm2_57_2.zip (12).

Research involving human participants, their data, or biological material

Policy information about studies with human participants or human data. See also policy information about sex, gender (identity/presentation), and sexual orientation and race, ethnicity and racism.

Reporting on sex and gender	No human participants were used in this study.
Reporting on race, ethnicity, or other socially relevant groupings	No human participants were used in this study.
Population characteristics	No human participants were used in this study.
Recruitment	No human participants were used in this study.
Ethics oversight	No human participants were used in this study.

Note that full information on the approval of the study protocol must also be provided in the manuscript.

Field-specific reporting

Please select the one below that is the best fit for your research. If you are not sure, read the appropriate sections before making your selection.

- [x] Life sciences
- [ ] Behavioural & social sciences
- [ ] Ecological, evolutionary & environmental sciences

For a reference copy of the document with all sections, see nature.com/documents/nr-reporting-summary-flat.pdf

Life sciences study design

All studies must disclose on these points even when the disclosure is negative.

Sample size	For experiments using publicly available data, sample sizes were chosen based on data availability, using the largest amount of data possible. For experiments based on data acquired for this study, small samples were used for demonstrations of the algorithms' performance and functionalities, with care taken to draw conclusions within the limits of what the data could reliably support, avoiding over-generalization or unwarranted extrapolation of findings.
Data exclusions	No data was excluded from the datasets used in this study.
Replication	The reproducibility of our experimental findings was ensured through the use of a fixed set of code applied to datasets. All analyses were conducted using this standardized algorithmic approach, allowing for consistent replication of results. Multiple test runs of our algorithms were performed on the same datasets during developments to confirm consistency in outputs. All code used and developed in this study is available in a public repository (https://github.com/HenriquesLab/SReD/). We documented all parameters and settings used in our algorithms to ensure transparency and facilitate exact replication.
Randomization	Sample randomization does not apply to our study because the entirety of each dataset was used in our analyses.
Blinding	Blinding procedures were not applicable to our study due to the nature of our research design and data analysis approach. Our study focused on the algorithmic analysis of complete datasets through automated computational processes.

Reporting for specific materials, systems and methods

We require information from authors about some types of materials, experimental systems and methods used in many studies. Here, indicate whether each material, system or method listed is relevant to your study. If you are not sure if a list item applies to your research, read the appropriate section before selecting a response.

Materials & experimental systems

n/a	Involved in the study	
☐	☒	Antibodies
☐	☒	Eukaryotic cell lines
☒	☐	Palaeontology and archaeology
☒	☐	Animals and other organisms
☒	☐	Clinical data
☒	☐	Dual use research of concern
☒	☐	Plants

Methods

n/a	Involved in the study	
☒	☐	ChIP-seq
☒	☐	Flow cytometry
☒	☐	MRI-based neuroimaging

Antibodies

Antibodies used	Mouse anti-CD3 (CD3 clone 17A2; BV711-conjugated, Biolegend), 200 µL per well at final concentration of 1µg/mL. Antibody produced at the Flow Cytometry & Antibodies Unit of Instituto Gulbenkian de Ciência, Oeiras, Portugal.
Validation	https://www.antibodyregistry.org/AB_2563945<br>ref: BioLegend Cat# 100241, RRID:AB_2563945

Eukaryotic cell lines

Policy information about cell lines and Sex and Gender in Research

Cell line source(s)	Jurkat cells (clone E6-1) provided by ATCC. RPE1-EB3-GFP cells provided by Dr. Monica Bettencourt-Dias (Instituto Gulbenkian de Ciência, Oeiras, Portugal)
Authentication	Cells were authenticated using microscopy to observe morphological and behavioural features.
Mycoplasma contamination	All cell lines cultured in our laboratory tested negative for mycoplasma contamination.
Commonly misidentified lines<br>(See ICLAC register)	No commonly misidentified lines.

Plants

Seed stocks	No plants were used in this study.
Novel plant genotypes	No plants were used in this study.
Authentication	No plants were used in this study.
