# Supplementary material for: Structural Repetition Detector for multi-scale quantitative mapping of molecular complexes through microscopy
Source: Nat Commun. 2025 Jul 1;16:5767. doi: 10.1038/s41467-025-60709-1 (PMC12219329; doi:10.1038/s41467-025-60709-1)
Supplement: Supplementary file 3 — Description of Additional Supplementary Files [file 41467_2025_60709_MOESM3_ESM.pdf]

Description of Additional Supplementary Files

Supplementary Movie 1. Representative SReD analysis pipeline for HIV particle detection in transmission electron microscopy data.
